# Tumour-derived Interleukin 35 promotes pancreatic ductal adenocarcinoma cell extravasation and metastasis by inducing ICAM1 expression

**DOI:** 10.1038/ncomms14035

**Published:** 2017-01-19

**Authors:** Chongbiao Huang, Na Li, Zengxun Li, Antao Chang, Yanan Chen, Tiansuo Zhao, Yang Li, Xiuchao Wang, Wei Zhang, Zhimin Wang, Lin Luo, Jingjing Shi, Shengyu Yang, He Ren, Jihui Hao

**Affiliations:** 1 Department of Pancreatic Cancer, Tianjin Medical University Cancer Institute and Hospital, National Clinical Research Center for Cancer, Key Laboratory of Cancer Prevention and Therapy, Tianjin 300060, China; 2 Senior Ward, Tianjin Medical University Cancer Institute and Hospital, National Clinical Research Center for Cancer, Key Laboratory of Cancer Prevention and Therapy, Tianjin 300060, China; 3 School of Medicine, Nankai University, Tianjin 300071, China; 4 Tianjin Hepingqu Gynaechology and Obstetrics Hospital, Tianjin 300000, China; 5 Department of Tissue Bank, Tianjin Medical University Cancer Institute and Hospital, National Clinical Research Center for Cancer, Key Laboratory of Cancer Prevention and Therapy, Tianjin 300060, China; 6 Department of Cellular and Molecular Physiology, Penn State University College of Medicine, Hershey, Pennsylvania 17033, USA

## Abstract

Interleukin 35 (IL-35) is a novel member of the IL-12 family, consisting of an EBV-induced gene 3 (EBI3) subunit and a P35 subunit. IL-35 is an immune-suppressive cytokine mainly produced by regulatory T cells. However, the role of IL-35 in cancer metastasis and progression is not well understood. Here we demonstrate that IL-35 is overexpressed in human pancreatic ductal adenocarcinoma (PDAC) tissues, and that IL-35 overexpression is associated with poor prognosis in PDAC patients. IL-35 has critical roles in PDAC cell extravasation and metastasis by facilitating the adhesion to endothelial cells and transendothelial extravasation. Mechanistically, IL-35 promotes *ICAM1* overexpression through a GP130-STAT1 signalling pathway, which facilitates endothelial adhesion and transendothelial migration via an ICAM1–fibrinogen–ICAM1 bridge. In an orthotopic xenograft model, IL-35 promotes spontaneous pancreatic cancer metastasis in an *ICAM1*-dependent manner. Together, our results indicate additional functions of IL-35 in promoting PDAC metastasis through mediating *ICAM1* expression.

## Abstract (web summary)

Interleukin-35 (IL-35) is an immune suppressive cytokine produced by T-cells. Here, the authors show that IL-35 is overexpressed by human pancreatic cancer cells and show, in a mouse model, that IL-35 promotes metastasis in an autocrine/paracrine manner via induction of ICAM-1 expression.

Pancreatic ductal adenocarcinoma (PDAC), one of the leading causes of cancer-related mortality, has a 5-year survival rate of <5% (ref. 1). The poor prognosis in PDAC is primarily due to early onset of distant metastasis^2^,^3^,^4^. Understanding the mechanisms that regulate PDAC metastasis are critical to improving PDAC treatment.

Both of the major theories of cancer metastasis—the seed and soil hypothesis and the mechanical trapping theory—view tumour cell adhesion to the endothelia as one of the key steps in the metastatic process^5^,^6^. Adhesion to endothelial membranes is the initial step in extravasation, which is followed by transendothelial migration^7^. Only a small proportion of the circulating tumour cells are thought to be able to extravasate into distant tissues and establish metastases^8^.

Interleukin 35 (IL-35) is a recently identified member of the IL-12 family of cytokines, which are primarily expressed by regulatory T cells (Tregs), such as natural Tregs and inducible Tregs^9^,^10^. Although it shares components with IL-12 and IL-27, IL-35 has distinctly different immunological functions. Instead of promoting an inflammatory response similar to those of other members of the IL-12 family, IL-35 exhibits potent immunosuppressive effects comparable to those of IL-10 and transforming growth factor-β^11^. The IL-35 receptor (IL-35R) is composed of IL-12Rβ2 and GP130. IL-35 can signal through the homodimers GP130:GP130 or IL-12Rβ2:IL-12Rβ2, as well as the heterodimer IL-12Rβ2:GP130 (ref. 12). After the engagement of IL-35R, IL-35 signalling is initiated by the activation of members of the Janus kinase family and then members of the signal transducer and activator of transcription (STAT) family are phosphorylated and translocated into the nucleus, where they initiate transcription of target genes^13^.

The EBI3 protein is frequently expressed in nasopharyngeal carcinoma^14^ and lung cancer^15^. Recently, Pylayeva-Gupta *et al*.^16^ have reported that IL-35 produced by B cells has a pro-tumourigenic role in pancreatic cancer through a mechanism involving the IL-35-mediated stimulation of tumour cell proliferation. However, the role of IL-35 in PDAC metastasis is not clear. Here we sought to investigate the clinical significance of IL-35 in pancreatic carcinoma tissues. We identified a crucial role of IL-35 in the extravasation and metastasis processes in human pancreatic ductal carcinoma.

## Results

### IL-35 expression in pancreatic tumour tissues and cell lines

**Figure 1:**
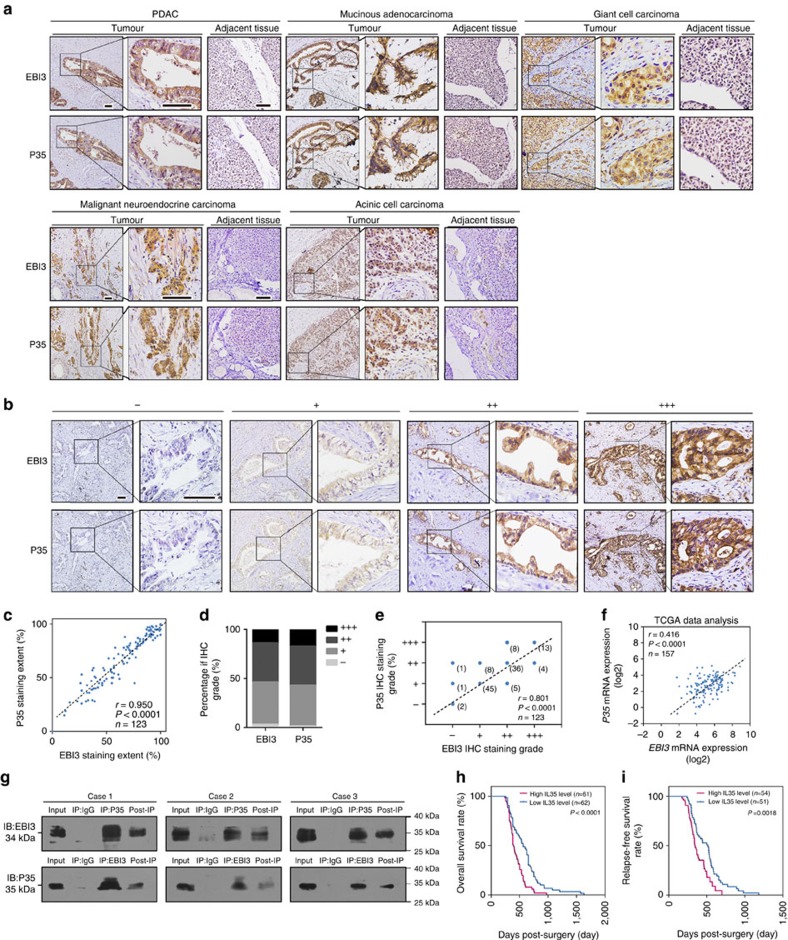
Expression of IL-35 and clinical significance in pancreatic carcinomas. Consecutive sections of pancreatic carcinoma tissues were used to analyse the expression levels of the two subunits of the IL-35 ligand: EBI3 and P35. (**a**) IHC staining of EBI3 and P35 proteins in five representative histologic types of pancreatic carcinoma (PDAC, mucinous adenocarcinoma, giant cell carcinoma, malignant neuroendocrine carcinoma and mucous cell carcinoma) and the adjacent normal pancreatic tissues. (**b**) Representative images are shown for absent, low, median and high expression of EBI3 and P35 in IHC staining in PDAC tissues. (**c**–**e**) The distribution of IHC results (**c**), staining extent correlation (**d**, Pearson's correlation analysis) and IHC result correlation (**e**, Spearman's correlation analysis) analysis of EBI3 and P35 in 123 PDAC patients. (**f**) Spearman's correlation analysis of the mRNA expression profiles of *EBI3* and *P35* in 157 PDAC patients from TCGA. (**g**) Co-immunoprecipitation and western blot analysis of EBI3 and P35 proteins from tumour tissue lysates of three PDAC patients, the remaining supernatant post immunoprecipitation (Post-IP) was also detected. (**h**,**i**) Kaplan–Meier analysis of OS (**h**) and RFS (**i**) of 123 PDAC patients (*P*=0.0010 and 0.0072, respectively, by log-rank test) according to different IL-35 levels. The test of significance is two-sided. Figure panel pairs in **a** and **b** represent images taken with different zooming options; scale bars, 100 μm.

As, to our knowledge, no specific antibodies for IL-35 are currently available, in this study we used the expression levels of EBI3 and P35 as a proxy for the level of IL-35. To investigate the expression pattern of IL-35 in pancreatic carcinoma, we performed immunohistochemistry (IHC) staining to detect EBI3 and P35 co-expression in tumour tissues and the adjacent normal pancreas tissues (pancreatic tissues 2–3 cm around the tumour border; Supplementary Fig. 1) of five histopathological subtypes of pancreatic carcinoma (PDAC, mucinous carcinoma, giant cell carcinoma, malignant neuroendocrine carcinoma and acinic cell carcinoma). EBI3 and P35 were frequently co-expressed in the cytoplasm of the five types of tumour tissue but they were rarely expressed in the adjacent normal tissues and the adjacent pre-neoplastic lesions (Fig. 1a and Supplementary Fig. 2).

To investigate the biological and clinicopathologic significance of IL-35 in pancreatic carcinomas, we performed immunohistochemical analysis of EBI3 and P35 in specimens from 123 patients with PDAC (Fig. 1b). Interestingly, the extent of staining (*r*=0.950, *P*<0.0001, Pearson's correlation analysis) and the final staining scores (*r*=0.801, *P*<0.0001, Spearman's correlation analysis) of EBI3 and P35 were strongly correlated (Fig. 1c–e). The correlation between *EBI3* and *P35* was further confirmed by the messenger RNA level in a cohort of 157 PDAC patients from The Cancer Genome Atlas (TCGA) (*r*=0.418, *P*<0.0001, Spearman's correlation analysis; Fig. 1f). As EBI3 forms IL-27 with P28 and P35 forms IL-12 with P40, we examined the expression levels of P28 and P40 in the PDAC tissues. As shown in Supplementary Fig. 3, no significant correlation between P35 and P40 or P28 and EBI3 was detected in PDAC (Spearman's correlation analysis), thus suggesting that EBI3 and P35 in PDAC tissues mainly exist in the form of IL-35. To more critically evaluate this possibility, we used antibodies to co-immunoprecipitate EBI3 and P35 in PDAC specimens. As shown in Fig. 1g and Supplementary Fig. 3E, the P35 antibody successfully immunoprecipitated EBI3 in PDAC tumour tissues and PDAC primary cell lines, and vice versa, whereas P28 and P40 antibodies immunoprecipitated much lower amounts of EBI3 and P35. More importantly, P35 immunoprecipitation depleted P35 and EBI3 proportionally in the post-IP cell lysate and vice versa, thus strongly suggesting that the P35 and EBI3 subunits in the PDAC tissues predominantly exist as IL-35 heterodimers. The expression of IL-35 and IL-35R were also detected in human PDAC cell lines at levels comparable to that in nTreg cells (Supplementary Fig. 4).

By using the co-expression level of EBI3 and P35 as a proxy for the IL-35 level, the PDAC samples were stratified into ‘IL-35 high (both EBI3 and P35 scored as ++/+++)' and ‘IL-35 low (other than IL-35 high)'. As shown in Table 1, a high expression of IL-35 was strongly correlated with advanced pathological TNM staging (*P*=0.003, *χ*^2^-tests), regional lymph node involvement (*P*=0.028, *χ*^2^-tests), poor differentiation (*P*=0.009, *χ*^2^-tests), blood vessel infiltration (*P*=0.003, *χ*^2^-tests) and tumour size (*P*=0.049, *χ*^2^-tests). The overall survival (OS) and relapse-free survival (RFS) times of PDAC patients with low IL-35 expression were significantly longer than those with high IL-35 staining (*P*<0.0001 and *P*=0.0018, respectively, log-rank test; Fig. 1h,i). Importantly, a multivariate Cox's regression analysis revealed that the IL-35 expression level was an independent prognostic factor for the OS (hazard ratio, 1.765; 95% confidence interval, 1.142–2.730; *P*=0.0110) of patients with PDAC (Table 2). Together, our data suggest a potential role of IL-35 in PDAC metastasis and progression.

### IL-35 facilitates PDAC endothelial adhesion and TEM *in vitro*

**Figure 2:**
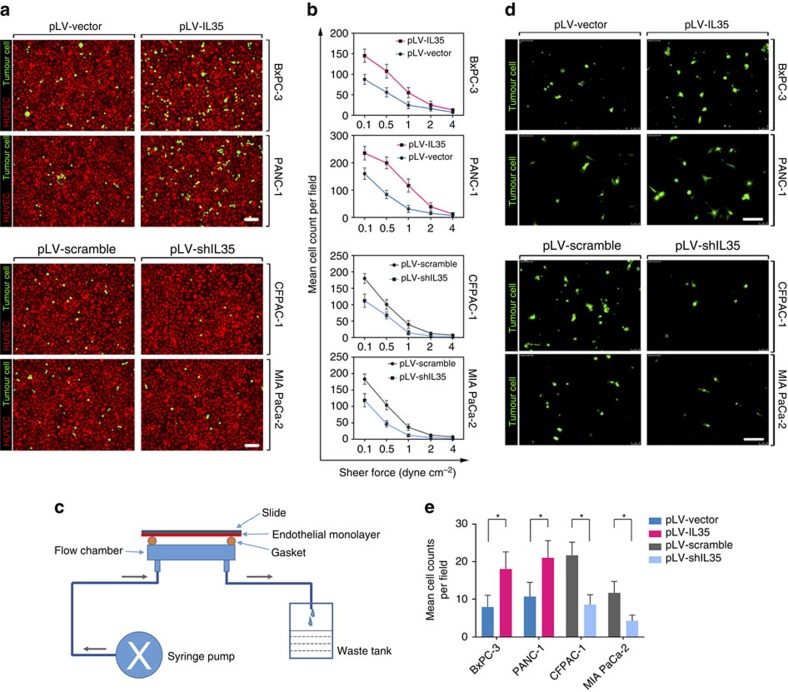
IL-35 facilitates PDAC cell endothelial adhesion and TEM *in vitro*. (**a**) Representative immunofluorescence images demonstrating the dynamic flow assays at the shear force of 1 dyne cm^−2^. (**b**) Adhesion-shear force curves of the adhesion assays performed at shear forces from 0.1 to 4 dyne cm^−2^. (**c**) Schematic diagram of the dynamic flow assay kit. (**d**,**e**) The indicated tumour cells were seeded on HUVEC monolayers reaching confluence in 24-well Transwell inserts. After incubation for 12 h, the transmigrated tumour cells were imaged. The experiments were replicated three times; shown are the means±s.d. Unpaired *t*-tests were used in **b** and **e**. Tests of significance are two-sided. *P<0.05. Scale bars, 100 μm.

To explore the roles of IL-35 in tumour progression, we established stable cell lines in which the IL-35 expression level was overexpressed or knocked down (Supplementary Fig. 5). The effects of IL-35 overexpression or knockdown on the tumour cell migration, invasion and proliferation were negligible (Supplementary Fig. 6). However, in monolayer dynamic adhesion assays, the overexpression of IL-35 significantly enhanced the PDAC cell adhesion to human umbilical vein endothelial cells (HUVECs) in a fibrinogen-dependent manner (unpaired *t*-tests). As shown in Fig. 2a–c, the IL-35 overexpression strongly enhanced the adhesion of BxPC-3 and PANC-1 cells to HUVECs in the presence of fibrinogen. In contrast, IL-35 knockdown significantly inhibited the fibrinogen-dependent HUVEC adhesion of MIA PaCa-2 and CFPAC-1 cells (unpaired *t*-tests). Similar results were observed in static endothelial adhesion assays (Supplementary Fig. 7). Given the important role of endothelial adhesion in transmembrane endothelial migration (TEM)^7^, we investigated the role of IL-35 in the transmigration of PDAC cells through the endothelial cells. As shown in Fig. 2d,e, the transendothelial migration of PDAC cells significantly increased when IL-35 was upregulated and decreased when IL-35 was downregulated in the presence of fibrinogen (unpaired *t*-tests).

We found that high IL-35 levels were associated with a high risk of regional lymph node involvement (Table 1). As lymphatic endothelia are quite different from the blood vessel endothelia, we especially detected the role of IL-35 in lymphatic endothelial adhesion. The results revealed that IL-35 significantly facilitated PDAC adhesion to lymphatic endothelial monolayers (unpaired *t*-tests), as it did in the HUVEC adhesion assays (Supplementary Fig. 8).

### ICAM1 mediates IL-35-induced endothelial adhesion and TEM

**Figure 3:**
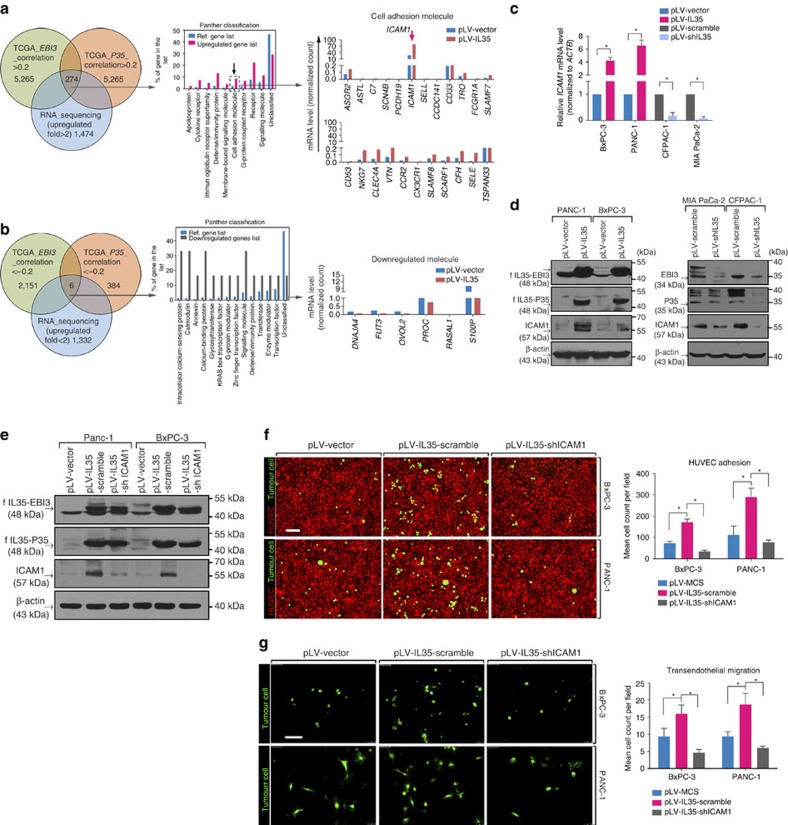
*ICAM1* is a novel IL-35 target gene critical for IL-35-induced endothelial adhesion and TEM. (**a**) Bioinformatics analysis of upregulated genes in genome-wide mRNA sequencing of IL-35-overexpressing PANC-1 cells versus control PANC-1 cells (left). The genes significantly correlated with *EBI3* and *P35* in the TCGA mRNA expression analysis were subjected to PANTHER classification (middle). The candidate cell adhesion genes are shown (right). (**b**) Bioinformatics analysis of downregulated genes as in **a**. (**c**,**d**) Cells were transfected with lentiviruses to upregulate or downregulate IL-35 expression. Forty-eight hours later, RT–PCR (**c**) and western blotting (**d**) were performed to verify the regulating effect of IL-35 on *ICAM1* in the indicated cell lines. (**e**) Western blotting assays to confirm stable ICAM1 knockdown in the indicated cell lines. (**f**) Endothelial adhesion assays of indicated cells performed as described in Fig. 2a. (**g**) TEM assays performed as described in Fig. 2d. Experiments were replicated three times; shown are the mean values±s.d. Unpaired *t*-test was used in **c**,**f** and **g**. Tests of significance are two-sided. *P<0.05. Scale bars, 100 μm.

To understand the molecular mechanism underlying IL-35-mediated endothelial cell adhesion, we performed genome-wide mRNA sequencing in PANC-1 cells with or without overexpression of IL-35. The mRNA sequencing data were verified by reverse transcription–PCR (RT–PCR) assays (Supplementary Fig. 9). Among the 17,733 sequenced genes, 1,474 were upregulated over 2-fold and 1,332 were downregulated over 2-fold. To narrow the range of the targets, we conjointly analysed our mRNA sequencing data and the mRNA expression profiles of PDAC patients from TCGA. As shown in Fig. 3a, among the 1,474 unregulated genes in the mRNA sequencing, only 274 were significantly correlated with both EBI3 and P35 in the TCGA data analysis (*r*>0.2, *P*<0.05, Spearman's correlation analysis). PANTHER classification analysis^17^ revealed that the proportion of cell adhesion molecules (2.44 versus 8.39%; *P*<0.0001) was significantly higher than expected, thus supporting our findings that IL-35 functions in the monolayer cell adhesion, beyond its well-known immunosuppressive effects. In addition, we also analysed the 1,332 downregulated genes. However, the six genes screened out were not cell adhesion molecules (Fig. 3b). In the 23 cell adhesion molecules, we identified that only *ICAM1*, a transmembrane molecule stabilizing cell–cell interactions and facilitating transendothelial transmigration^18^,^19^, was abundantly expressed in PDAC cells and significantly regulated by IL-35 in PDAC cells (unpaired *t*-tests; Fig. 3c,d and Supplementary Fig. 10).

**Figure 4:**
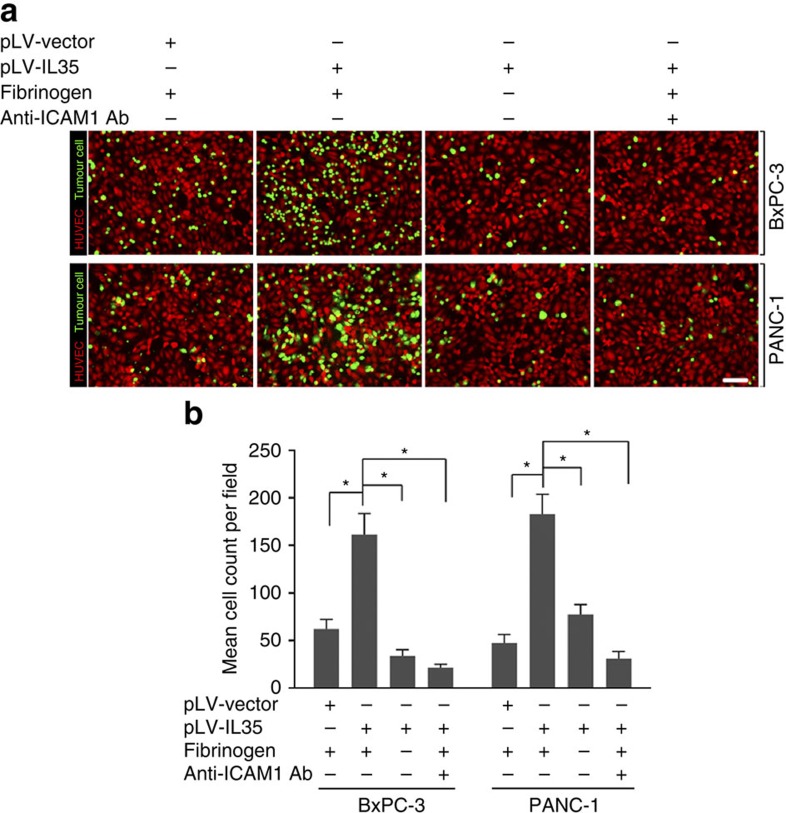
ICAM1 facilitates adhesion to HUVECs via the ICAM1–fibrinogen–ICAM1 bridge. (**a**,**b**) HUVECs stained with a CytoPainter Cell Tracking Staining Kit (red) were pretreated with or without anti-ICAM1 antibody (10 μg ml^−1^) for 30 min. Calcein AM-labelled (green) IL-35-overexpressing or control tumour cells were added and then co-cultured with or without fibrinogen (0.88 μM) for 1 h. The adhered tumour cells and HUVECs were captured by a fluorescence microscope (**a**). The average adhered tumour cells of five random fields of × 100 magnification were calculated (**b**). Experiments were replicated three times; shown are the mean values±s.d. Unpaired *t*-test was used; tests of significance are two-sided. *P<0.05. Scale bars, 100 μm.

To examine whether the IL-35-induced endothelial adhesion and TEM were due to the IL-35-ICAM1 axis, we stably knocked down *ICAM1* expression in PANC-1 and BxPC-3 cells stably overexpressing IL-35 (Fig. 3e). As shown in Fig. 3f,g, ICAM1 knockdown in PDAC cells abrogated the IL-35-mediated endothelial cell adhesion and TEM. Conventionally, ICAM1 mediates the adhesion of leukocytes to endothelial cells via binding to integrin molecules^20^. However, two ICAM1 molecules on opposing cells can be bridged by fibrinogen, thereby inducing leukocyte–endothelium adhesion^21^. Given that the IL-35-induced adhesion to HUVECs is fibrinogen-dependent, we hypothesized that IL-35 promotes tumour–endothelial cell adhesion through an ICAM1–fibrinogen–ICAM1 bridge. This possibility was confirmed by the finding that the pre-incubation of HUVEC cells with ICAM1 blocking antibody also abrogated the IL-35-mediated adhesion of PDAC cells (Fig. 4). Together, our data suggest that IL-35 overexpression in PDAC promotes ICAM1 expression, which in turn facilitates adhesion to endothelial cells through an ICAM1–fibrinogen–ICAM1 bridge.

**Figure 5:**
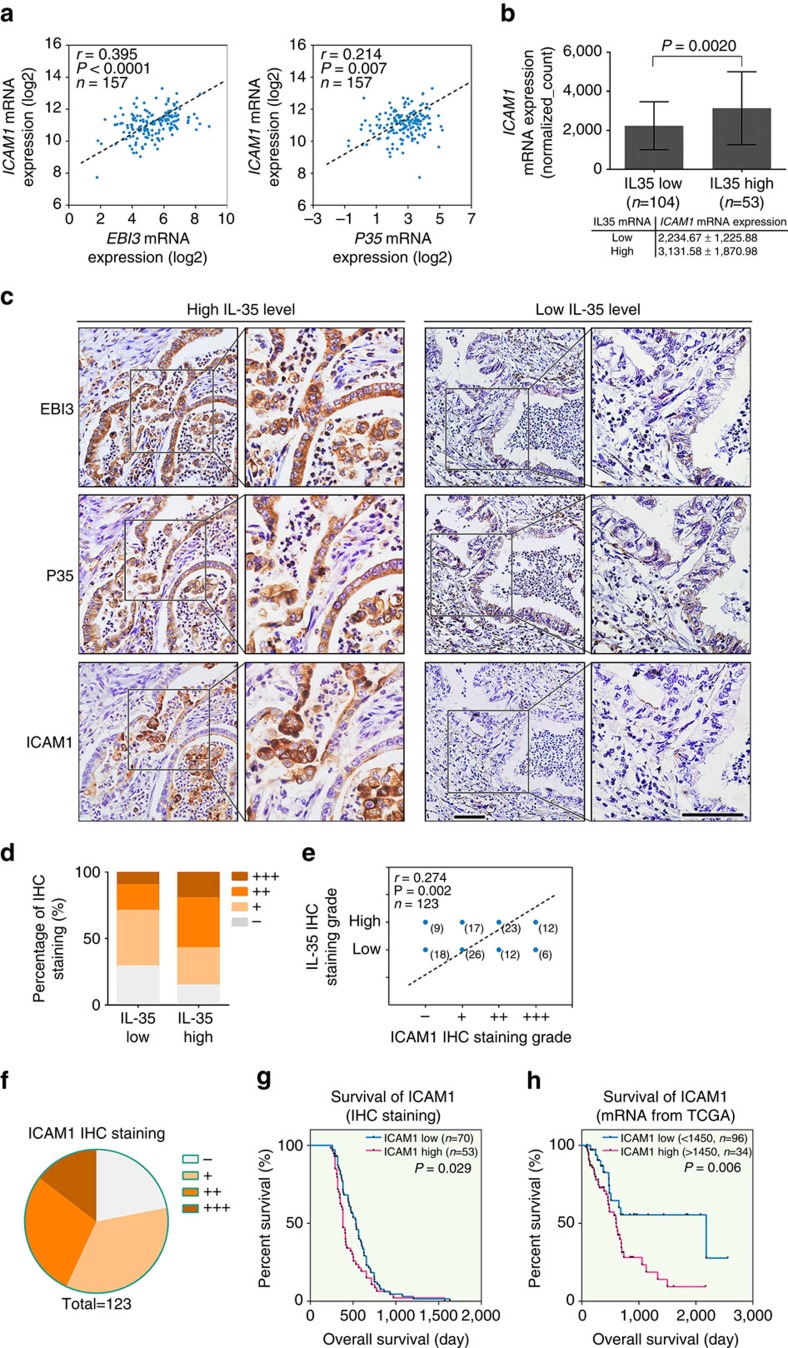
Correlation between *ICAM1* and IL-35 expression in human PDAC tissues. (**a**) Spearman's correlation analysis of the mRNA expression profiles of *EBI3* and *ICAM1* (left), as well as *P35* and *ICAM1* (right), in 157 PDAC patients from TCGA. (**b**) The 157 patients were stratified into two groups on the basis of the mRNA level of *EBI3* and *P35*: IL-35 high, both *EBI3* and *P35* mRNA levels higher than median; others were IL-35 low. The *ICAM1* mRNA levels of the two groups are presented. Unpaired *t*-tests were used; shown are the mean values±s.d. (**c**) Representative IHC staining images of high and low levels for ICAM1, EBI3 and P35 in consecutive sections are shown. Figure panels pairs represents images taken with different zooming options; scale bars, 100 μm. (**d**) Real distribution of IHC results between ICAM1 and IL-35 expression. (**e**) Spearman's correlation analysis of ICAM1 and IL-35 IHC staining results in 123 human PDAC surgical samples. (**f**) Real distribution of immunohistochemical results of ICAM1 in 123 PDAC tissues. (**g**) Kaplan–Meier analysis of OS of 123 PDAC patients according to different ICAM1 levels by IHC staining (ICAM1 low, −/+; ICAM1 high, ++/+++). (**h**) mRNA profiles and follow-up data of 130 PDAC patients from TCGA analysed for the correlation of the *ICAM1* mRNA expression and survival. Log-rank tests were used in **g** and **h**. Tests of significance are two-sided; *P<0.05.

Subsequently, the *in vivo* relationship between IL-35 and ICAM1 was explored. First, the data from TCGA were analysed. As shown in Fig. 5a,b, the *ICAM1* mRNA level in IL-35 high patients was significantly higher than that in IL-35 low patients (3131.58±1870.98 versus 2234.67±1225.88; *P*=0.002, unpaired *t*-test), thus suggesting that IL-35 promotes *ICAM1* expression in PDAC. Next, we examined the correlation between the protein levels of IL-35 and ICAM1 by immunohistochemical staining in a cohort of 123 PDAC specimens. As shown in Fig. 5c, the ICAM1 expression co-localized with EBI3 and P35 in consecutive sections of the PDAC tissues. The IL-35 expression level in PDAC tissues was significantly associated with the ICAM1 expression level (*r*=0.274, *P*=0.0020, Spearman's correlation analysis), that is, tumour tissues with a high IL-35 level usually had a high ICAM1 expression (Fig. 5d,e). These data present solid evidence that IL-35 plays a critical role in inducing *ICAM1* overexpression in PDAC tissues. In addition, we found that the expression level of *ICAM1* was also negatively associated with the survival of PDAC patients (Fig. 5g,h).

### IL-35 induces *ICAM1* expression by one branch of its pathway

**Figure 6:**
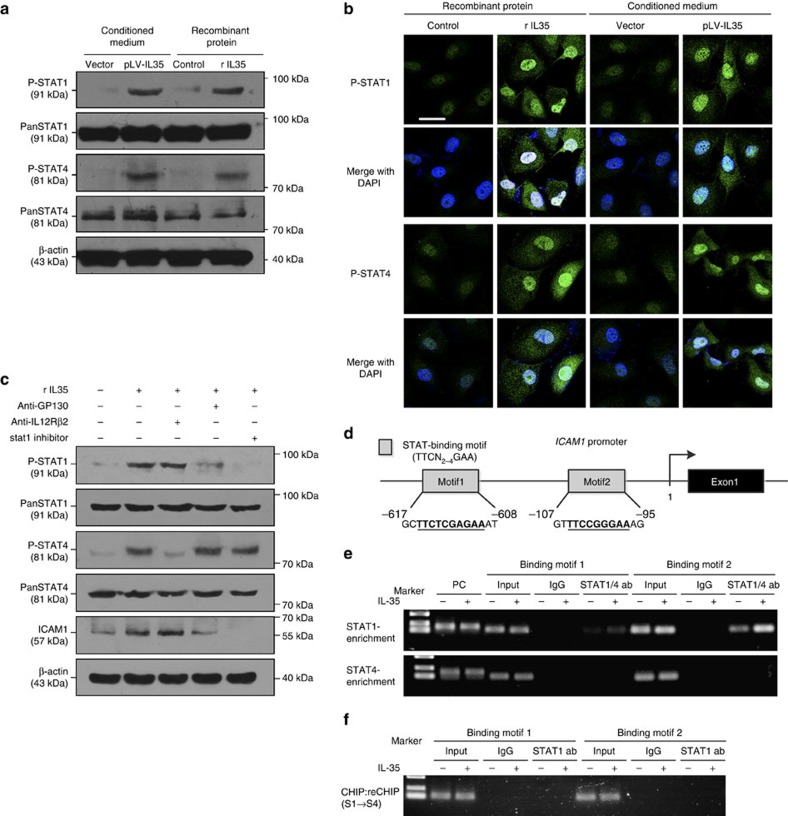
IL-35 regulates *ICAM1* expression via phosphorylated STAT1 homodimer. (**a**) PANC-1 cells treated with conditioned medium or recombinant IL-35 (rIL-35) for 30 min, probed with antibodies to phosphorylated (-p) STAT1 and STAT4. (**b**) Representative immunofluorescence images demonstrate the translocation process of p-STAT1 and p-STAT4 to the nucleus in PANC-1 cells incubated with conditioned medium or recombinant IL-35 for 1 h. Scale bars, 30 μm. (**c**) PANC-1 cells were pretreated with or without the STAT1 inhibitor fludarabine (2 μg ml^−1^), anti-GP130 antibody (100 ng ml^−1^) and anti-IL12Rβ2 antibody (2 μg ml^−1^) for 30 min, then cultured with recombinant IL-35 (100 ng ml^−1^) for 16 h and subjected to western blotting assays. (**d**) Human *ICAM1* promoter; two of the identified STAT-binding motifs were investigated. (**e**) CHIP analysis of PANC-1 cells pretreated with or without IL-35 (100 ng ml^−1^) for 90 min. Chromatin was immunoprecipitated with anti-STAT1 or anti-STAT4 antibodies and then subjected to RT–PCR analysis. (**f**) CHIP: reCHIP analysis of PANC-1 cells prepared as in **e**; Chromatin was first immunoprecipitated with anti-STAT1 antibody and then immunoprecipitated again with anti-STAT4 (S1→S4).

IL-35 is unique from other cytokines of the IL-12 family in that it can induce a downstream signalling cascade through a GP130:IL-12Rβ heterodimer or through a GP130:GP130 and/or IL-12Rβ2:IL-12Rβ2 homodimer. The binding of IL-35 to homodimeric receptors activates only one branch of the signal transduction cascade (that is, phosphorylated (-p) STAT1 or p-STAT4)^12^. As shown in Fig. 6a,b, the treatment of PDAC cells with IL-35 led to the increased phosphorylation of STAT1 and STAT4, and the nuclear translocation of p-STAT1 and p-STAT4, a result consistent with previous findings in T cells^13^.

To investigate the mechanism by which IL-35 regulates *ICAM1* expression, we used a specific antibody to block GP130 or IL-12Rβ2 on PANC-1 cells. GP130 blockage inhibited the phosphorylation of STAT1 but not STAT4 and remarkably inhibited IL-35-mediated ICAM1 overexpression. In contrast, IL-12Rβ2 inhibition with blocking antibody inhibited the phosphorylation of STAT4 but not STAT1 and had no effect on ICAM1 expression (Fig. 6c). The essential role of STAT1 in IL-35-mediated ICAM1 expression was further confirmed with the STAT1 activation inhibitor fludarabine (Fig. 6c). Together, these results suggest that IL-35 stimulate *ICAM1* expression through only the STAT1 branch of the signalling cascade.

*In silico* analysis of the *ICAM1* promoters with a proposed consensus STAT-binding motif (TTC-N_2–4_-GAA, where ‘N_2–4_' indicates a range of two to four residues of any nucleotide) identified two potential binding sites^22^ (Fig. 6d). To determine whether IL-35 directly regulates *ICAM1* transcription through STAT1, we carried out chromatin immunoprecipitation (ChIP) and ChIP:reChIP analysis, to determine the binding of STAT1 to the *ICAM1* promoter. Primers were designed to investigate these sites through ChIP assays with anti-STAT1 and anti-STAT4 antibodies, respectively. As shown in Fig. 6e, IL-35 induced marked binding of STAT1 to both binding sites; however, no binding of STAT4 to either site was observed.

This result indicates that IL-35 uses the homodimer p-STAT1:p-STAT1 to induce *ICAM1* transcription. To examine the possibility that p-STAT1:p-STAT4 might also induce *ICAM1* expression, we performed a ‘ChIP:reChIP' analysis, in which the product of ChIP with anti-STAT1 antibody was subjected to a second ChIP with anti-STAT4 antibody. As expected, IL-35 did not induce binding of the STAT1–STAT4 heterodimer to either site (Fig. 6f). These results indicate the involvement of a STAT1–STAT1 homodimer as a unique biochemical effector of IL-35-ICAM1 signalling.

### GP130 is essential to the IL-35-associated poor prognosis

**Figure 7:**
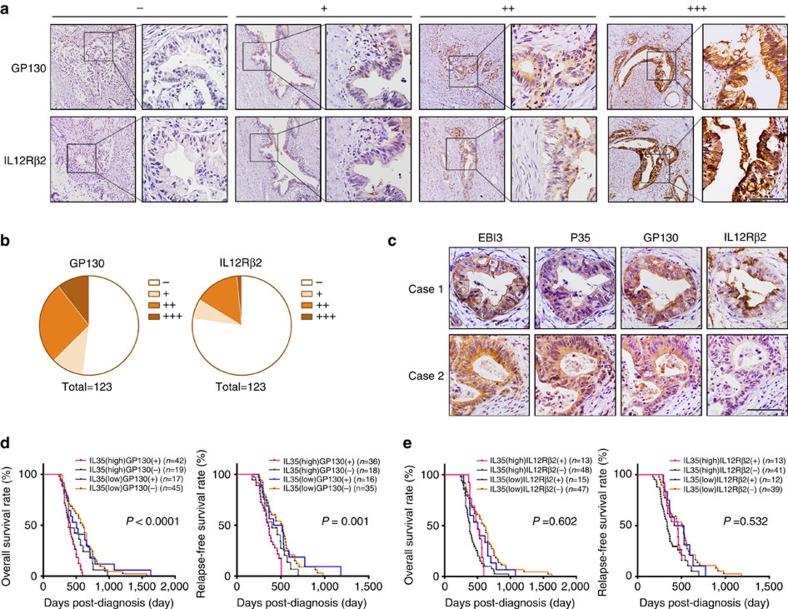
GP130 expression is essential to the IL-35-associated poor prognosis of patients with PDAC. (**a**) Examples are shown for absent, low, median and high expressions of GP130 and IL-12Rβ2 in PDAC tissues. Figure panel pairs represents images taken with different zooming options; scale bars, 100 μm. (**b**) Real distribution of IHC results of GP130 and IL-12Rβ2. (**c**) Examples are shown for the co-expression of IL-35 and IL-35R in PDAC tissues; scale bars, 100 μm. (**d**) Kaplan–Meier analysis of OS (left) and RFS (right) of 123 PDAC patients according to the expression levels of IL-35 and GP130. (**e**) Kaplan–Meier analysis as in **d** but according to the expression levels of IL-35 and IL-12Rβ2. Log-rank tests were used in **d** and **e**.

We further examined the role of the GP130 signalling branch in the IL-35-mediated PDAC progression. As shown in Fig. 7a, GP130 and IL-12Rβ2 were positively detected in the immunohistochemical staining of PDAC specimens. The expression patterns of these two proteins were different (Fig. 7b). Moreover, we found that the IL-35 ligand and IL-35R were frequently co-expressed in PDAC tissues (Fig. 7c). We divided the samples into two groups on the basis of the IL-35R expression level: IL-35R (+) and IL-35R (−). Then, the 123 PDAC cases were classified into four groups according to the expression levels of IL-35 and IL-35R: IL-35 (high) IL-35R (+), IL-35 (high) IL-35R (−), IL-35 (low) IL-35R (+) and IL-35 (low) IL-35R (−). Statistical analysis revealed a significant association between the expression levels of IL-35 and IL-35R (*r*=0.366, *P*<0.0001, Spearman's correlation analysis; Supplementary Table 3). These results suggest that in PDAC tissues, the IL-35 ligand is usually accompanied by the IL-35R, thus supporting our hypothesis that IL-35 facilitates PDAC progression by directly acting on tumour cells in an autocrine or paracrine manner.

Given that IL-35 induces ICAM1 expression only via the GP130-STAT1 signalling pathway, we subsequently explored the roles of the GP130 subunit in PDAC tissues. The 123 PDAC cases were stratified into four groups: IL-35 (high) GP130 (+), IL-35 (high) GP130 (−), IL-35 (low) GP130 (+) and IL-35 (low) GP130 (−). Patients with a high IL-35 level and positive GP130 staining had the poorest OS and RFS (Fig. 7d). The role of the IL-12Rβ2 subunit was also explored as GP130. However, no significant differences in OS or RFS were observed between the IL-12Rβ2-positive group and the IL-12Rβ2-negative group (log-rank test; Fig. 7e). These results suggest that in PDAC tissues, the IL-35-related poor prognosis is associated with the expression of GP130 but not IL-12Rβ2, a result consistent with our finding that IL-35 regulates ICAM1 expression only through the GP130:GP130 homodimer.

### IL-35 facilitates PDAC extravasation and metastasis in mice

To investigate whether IL-35 promotes extravasation *in vivo*, we injected Pan02 cells with mouse *IL-35* overexpression or knockdown, or *Icam1* knockdown (Supplementary Fig. 11) via the portal vein into mice. Severe combined immunodeficiency (SCID) mice were used to eliminate the disturbance caused by IL-35-related immunosuppressive effects, which may contribute to metastasis.

**Figure 8:**
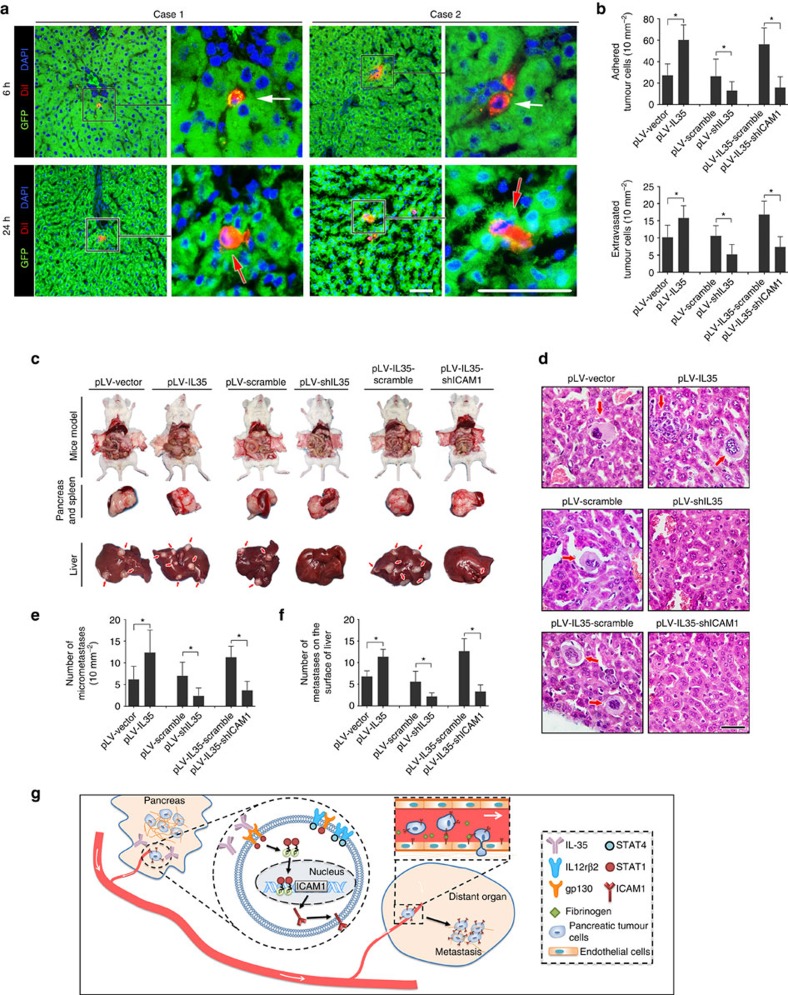
IL-35 facilitates PDAC cell extravasation and metastasis *in vivo* via upregulating ICAM1 expression. (**a**,**b**) DiI-labelled PDAC cells were injected into green fluorescent protein-knockin mice through the portal veins. At the time points of 6 and 24 h, animals were killed and livers were sliced. (**a**) Representative images of adhered/arrested tumour cells (white arrow) and extravasated tumour cells (red arrow) in livers are shown (*n*=6 for each group). (**b**) Numbers of adhered/arrested tumour cells and extravasated cells are analysed. (**c**–**f**) Indicated tumour cells were orthotopically transplanted into the SCID mouse pancreases to develop tumours (*n*=8 for each group). Representative images of the tumours and metastatic sites of livers are shown in **c**. Hematoxylin and eosin staining images of liver micrometastasis sites are shown in **d**. The total numbers of visible metastatic lesions in **c** and invisible micrometastases in **d** are presented in **e** and **f**, respectively. (**g**) Schematic diagram of pancreatic carcinoma undergoing a metastatic progression mediated by the IL-35-ICAM1 axis. After being secreted by pancreatic tumour cells, IL-35 acts on the tumour cells in an autocrine or paracrine manner. After binding to the homodimer GP130-GP130, IL-35 activates the Jak-STAT signalling pathway. STAT1 is phosphorylated and forms the p-STAT1/p-STAT1 homodimer. Then, the homodimers are transferred into the nucleus, where they activate the transcription of the *ICAM1* gene. The ICAM1 protein on the tumour cell surface enhances the extravasation process by facilitating adhesion to the endothelial cells and the transendothelial migration process in an ICAM1–fibrinogen–ICAM1 manner. The elevated extravasation activity promotes metastasis in distant organs or tissues. Unpaired *t*-tests were used in **b**,**e** and **f**; tests of significance are two-sided; shown are the mean values±s.d.; *P<0.05. Figure panel pairs in **a** represents images taken with different zooming options; scale bar, 100 μm (**a**) and 50 μm (**d**).

At the time points of 6 and 24 h after injection, the livers were harvested and analysed for adhered/arrested tumour cells and extravasated tumour cells, respectively. We observed that the overexpression of IL-35 greatly increased the number of adhered/arrested tumour cells and extravasated tumour cells, whereas the knockdown of IL-35 resulted in significantly decreased numbers of adhered/arrested tumour cells and extravasated tumour cells (unpaired *t*-test). In contrast, when ICAM1 was knocked down in IL-35-overexpressing Pan02 cells, the IL-35-induced adhesion and extravasation were abrogated (Fig. 8a,b). These data indicate that ICAM1 is essential for IL-35-mediated endothelial adhesion and extravasation.

We next evaluated the role of IL-35 in PDAC metastasis in an orthotopic pancreatic cancer mouse model by transplanting Pan02 into the pancreases of SCID mice. Pan02 cells developed primary tumours in the pancreas and distant metastases in the liver, mesenterium and peritoneum over the course of 6 weeks (Supplementary Fig. 12). We observed that the overexpression of IL-35 led to significantly increased numbers of visible metastases (6.80±1.30 versus 11.40±1.67; *P*=0.0013, unpaired *t*-test) and invisible metastases (6.20±1.36 versus 12.40±2.32; *P*=0.0496, unpaired *t*-test) in the liver. Meanwhile, the downregulation of IL-35 resulted in a decrease in visible metastases (5.60±2.41 versus 2.20±0.84; *P*=0.0175, unpaired *t*-test) and invisible metastases (7.00±1.41 versus 2.40±0.81; *P*=0.0225, unpaired *t*-test). The increase in metastases induced by IL-35 was abolished after ICAM1 was knocked down (Fig. 8c–f). Together, these results suggest that IL-35 plays a critical role in the extravasation and metastasis of PDAC *in vivo*.

## Discussion

Cytokines in the tumour microenvironment play important roles in tumour carcinogenesis, angiogenesis, metastasis and other malignant behaviours^23^. In addition to stromal cells and infiltrating immune cells, tumour cells also produce and secrete various types of cytokines, the most widely known of which are tumour necrosis factor-α^24^, transforming growth factor-β^25^ and IL-10 (refs 26, 27).

IL-35 is a novel member of the IL-12 family, which was identified in 2007 (ref. 11). Two substantial immunological functions of IL-35 have been reported: suppressing T-cell proliferation and converting T cells into suppressive inducible Tregs^10^. Although IL-35 has been implicated in many pathological processes such as autoimmune diseases, understanding of the role of IL-35 in cancer is very limited. A major challenge in the study of IL-35 is that no conformational antibodies specific to IL-35 are presently available. Several studies have explored the expression of IL-35 in several types of tumours with antibodies specific to EBI3 (refs 28, 29). However, whereas EBI3 can exist as free units^30^, and EBI3 and P35 are also subunits of IL-27 and IL-12, respectively, which are also expressed in tumour cells^31^,^32^, antibodies specific to either EIB3 or P35 are unsuitable for the detection IL-35.

In this study, we use the co-expression of the two IL-35 subunits EBI3 and P35 in consecutive sections as a proxy, to determine the IL-35 levels. We discovered that the EBI3 and P35 expression levels in PDAC tissues were highly correlated, thus suggesting that these two subunits in PDAC mainly exist in the form of IL-35. The EBI3 subunit co-immunoprecipitated with the P35 subunit and, more importantly, proportionally depleted the P35 subunit in the supernatant and vice versa. In addition, we analysed the expression levels of P28 and P40 with EBI3 and P35, to exclude the potential interference in which IL-27 and IL-12 were simultaneously expressed in these tissues. The statistical analysis revealed that there were no significant associations between the expressions of P40 and P35 or between P28 and EBI3 in PDAC tissues (Spearman's correlation analysis). These results strongly support the notion that EBI3 and P35 in PDAC tissue mainly exist in the form of L-35. As a result, the co-expression of EBI3 and P35 could be used as a surrogate for the IL-35 levels in PDAC.

We found that IL-35 overexpression was significantly associated with advanced TNM stage, nodal involvement, blood vessel infiltration and poor prognosis in PDAC patients (*χ*^2^-tests), thus suggesting a role of IL-35 in PDAC progression and metastasis. As a suppressive cytokine, IL-35 has been suggested to promote tumour growth via enhancing myeloid cell accumulation, tumour angiogenesis and suppression of tumour immunity^28^. In this study, we revealed a novel function of IL-35: enhancing the endothelial adhesion and TEM activity of PDAC cells *in vitro* and *in vivo*.

To investigate the mechanism of the IL-35-mediated endothelial adhesion and TEM, we analysed the mRNA sequencing data of cell lines and the mRNA expression profiles of PDAC patients from TCGA. The mRNA sequencing of the cell lines identified hundreds of downstream genes of IL-35; however, only molecules statistically correlated with IL-35 may be solidly regulated by IL-35 and play key roles in IL-35-induced tumour progression. Nevertheless, a significant correlation (Pearson's correlation analysis) between the candidate genes, and EBI3 and P35 in the TCGA analysis may not necessarily represent high expression levels in tumour cells, because in the tumour tissue sequenced by TCGA, a large proportion comprised stromal cells such as infiltrating immune cells. Therefore, the expression abundance of the candidate genes in tumour cells also needs to be considered. In the 23 candidate cell adhesion genes, only ICAM1 fulfilled this requirement.

The endothelial adhesion of tumour cells is mediated by specific molecules, such as ICAM1, VCAM1 and VAP1 (ref. 7). The interaction of the receptors on the tumour cells and endothelia can be bridged by integrins, selectins and platelets. In addition to the two integrins, macrophage adhesion ligand-1 (CD11b/CD18)^33^ and leukocyte function-associated antigen-1 (CD11a / CD18)^34^, fibrinogen is another important receptor of ICAM1 (ref. 21). Interestingly, because fibrinogen has a high affinity in bridging the two ICAM1 molecules, ICAM1–FIB–ICAM1 binding strongly enhances leukocyte–endothelium adhesion^21^. The important role of ICAM1–fibrinogen binding in cell–cell adhesion has been further confirmed by a recent work in which ICAM1–fibrinogen has been found to be an important mediator of platelet–monocyte interactions^35^. In the present study, we found that the IL-35-mediated adhesion to endothelial cells was dependent on ICAM1 and fibrinogen, thus suggesting that IL-35 mediates PDAC cell endothelial adhesion by facilitating ICAM1–fibrinogen–ICAM1 bridge formation^21^. Indeed, the blockage of ICAM1 in HUVECs leads to decreased adhesion activity, thus lending further support to this hypothesis. Given the key roles of ICAM1–fibrinogen binding in IL-35-mediated interaction, IL-35 might also promote PDAC adhesion to other membranes expressing ICAM1, such as the lymphatic endothelium^36^,^37^ and mesothelium^38^,^39^. As expected, we found that IL-35 also greatly enhanced the PDAC adhesion to the lymphatic endothelium *in vitro*. This enhanced adhesion may be the underlying mechanism by which a high IL-35 level is linked to a high risk of lymph node involvement. In addition, IL-35 significantly enhanced the adhesion of PDAC cells to mesothelial monolayers (unpaired *t*-test; Supplementary Fig. 13A–D). Interestingly, this result was consistent with our findings in the mice model: an elevation of IL-35 also resulted in elevated metastasis to the gut and mesentery (Supplementary Fig. 13E,F).

In addition to indirectly facilitating the TEM activity by promoting endothelial adhesion, ICAM1 has been reported to directly influence the TEM process^40^. In addition, ICAM-1 signalling recruits inflammatory immune cells such as macrophages and granulocytes^41^, thus leading to carcinogenesis^42^. IL-35 may also induce ICAM1 expression in immune cells or endothelial cells and influence the tumour immune microenvironment. Future investigation into the roles of the IL-35-ICAM1 signalling pathways is warranted.

Interestingly, although both subunits of IL-35R were expressed in PDAC cells, only GP130 was indispensable in IL-35-mediated ICAM1 expression. IL-35 leads to the phosphorylation of both STAT1 and STAT4, but only the pSTAT1–pSTAT1 homodimer can bind to the ICAM1 promoter and induce its transcription. This result has been supported by the work of Professor Vignali: the signalling of IL-35 can be transmitted by only one branch of its signal transduction cascades^13^. The critical role of GP130 in the IL-35-mediated PDAC progression was further confirmed in the survival analysis of PDAC patients, thus suggesting that IL-35 overexpression promotes PDAC metastasis through a GP130–STAT1 signalling pathway. In the orthotopic transplantation mouse model, we used the number of visible metastases in the liver to assess the hepatic metastasis status. Although this method is frequently used to analyse the metastasis process of PDAC in the liver^43^,^44^,^45^, the possibility that tumour cells directly disseminate to the liver during orthotopical injection could not be completely excluded. Thus, we also analysed the invisible micro-metastases inside the liver to assess the hepatic metastasis.

Our results suggested that blocking of the IL-35-ICAM1 signalling axis may reduce the metastasis risk in patients with PDAC. The IL-35 signalling could potentially be blocked by targeting the IL-35 ligand, IL-35R, STAT activation and ICAM1 protein. Blocking antibodies and small molecules for these targets might be useful in the treatment of metastatic PDAC. Several limitations to the present study are noted. First, we knocked down the IL-35 level by simultaneously inhibiting the EBI3 and P35 expression, to explore the role of IL-35 under basal conditions. However, the depletion of EBI3 and P35 may also have reduced the levels of IL-12 and IL-27, which might have been partially responsible for the biological effects in these experiments. Second, because no conformational antibodies specific to IL-35 are currently available, in the IHC staining study we relied on detecting the co-expression of EBI3 and P35. This method is inconvenient to use and the result is somewhat indirect. Third, in this study, we only retrospectively analysed a relatively small number of clinical samples. A prospective analysis of a larger number of samples would be important to verify the clinical significance of IL-35 before initiating clinical exploration. Finally, because IL-35 is not expressed in only pancreatic cancer, it may also promote the metastasis process of other cancers in an ICAM1-dependent pathway. Once this hypothesis is confirmed by further studies, the potential significance and usefulness of IL-35 in anti-tumour treatment would be greatly extended.

## Methods

### Cell culture and human sample collection

Human PDAC cell lines CFPAC-1, BxPC-3 and Panc-1 were obtained from the Type Culture Collection Committee of the Chinese Academy of Sciences (Shanghai, China), and MIA-PaCa-2 was obtained from the American Type Culture Collection. The cell lines were obtained in 2013 and authenticated in August 2014 through the short tandem repeat analysis method. The primary PDAC cell lines PTX0001, PTX0015, PTX0037 and PTX 0049 were purchased from WuXi Pharmatech Company (WuXi, China). Mycoplasma contamination was excluded in these cell lines. These cells were cultured in DMEM, RPMI-1640 or IMDM basic medium supplemented with 10% fetal bovine serum (FBS) at 37 °C in a humidified atmosphere of 95% air and 5% CO_2_.

A total of 123 sequential PDAC tissues and 15 other pathotypes of pancreatic tumour tissues and the adjacent pancreas tissues were collected from patients who had received radical surgery in 2010 at Tianjin Medical University Cancer Institute and Hospital (China). Retrospective clinicopathological data of these patients were also obtained, including age, sex, tumour size, regional lymph node status, TNM stage, pathologic type and differentiation. Among the 123 cases, only 105 had detailed RFS data.

The usage of these specimens and the patient information were approved by the Ethics Committee of the Tianjin Medical University Cancer Institute and Hospital. All patients provided written consent for the use of their specimens and disease information for future investigations according to the ethics committee.

### IHC and immunofluorescence

IHC was used to detect EBI3, P35, GP130, IL-12Rβ2, P28, P40 and ICAM1 expression in pancreatic tumour tissues. In brief, paraffin-embedded sections of PDAC were deparaffinized and then heated in a pressure pot for 3 min to retrieve the antigens. Then, the sections were incubated with primary antibodies (Supplementary Table 1) overnight at 4 °C. Antibody binding was detected using a peroxidase-conjugated secondary antibody at 37 °C for 30 min. A DAB Substrate Kit was used to perform the chromogenic reaction. The intensity of the staining was evaluated by using the following criteria: 0, negative; 1, low; 2, medium; and 3, high. The extent of staining was scored as 0, 0% stained; 1, 1–25% stained; 2, 26–50% stained; and 3, 51–100% stained. Five random fields (× 20 magnification) were evaluated under a light microscope. The final scores were calculated by multiplying the scores of the intensity with those of the extent and dividing the samples into four grades: 0, negative (−); 1–2, low staining (+); 3–5, medium staining (++); and 6–9, high staining (+++). The following criteria were used to quantify the expression levels of IL-35 in PDAC tissues: high expression, both EBI3 and P35 were scored as ++/+++; low expression, other than high expression.

Immunofluorescence staining was performed on the PDAC cell lines. Briefly, PANC-1 cells seeded on coverslips were cultured with IL-35 recombinant protein (100 ng ml^−1^) or conditioned medium (50%) for 60 min and then incubated with anti-p-STAT1 or anti-p-STAT4 antibodies at 4 °C overnight. Then, the cells were incubated with fluorescent dye-labelled secondary antibodies at room temperature for 1 h. The cells were again incubated with anti-fade 4,6-diamidino-2-phenylindole solution (1:1,000) and images were captured with a confocal fluorescence microscope. In another experiment, five types of PDAC cells were incubated with antibodies to detect the expression and localization of EBI3, P35, GP130 and IL-12Rβ2.

### Enzyme-linked immunosorbent assay

A human IL-35 ELISA kit (BioLegend) was used to detect the secretory IL-35 level in the supernatants of the cell cultures. According to the manufacturer's instructions, this ELISA kit uses the double-antibody sandwich technique (anti-EBI3 and anti-P35 antibodies) to detect the human IL-35 level.

### Monolayer adhesion assay

HUVECs were isolated from fresh human umbilical cords by digestion with 0.1% collagenase II and were cultured in Endothelial Cell Growth Medium (Lonza). Mesothelial cells were isolated from fresh human omentum by digestion with 0.25% trypsin and cultured in 1,640 medium supplemented with 10% FBS^46^.

A parallel plate flow chamber kit (GlycoTech) was used in the dynamic adhesion assay. Similar methods have previously been described elsewhere^47^. HUVECs, mesothelial cells or lymphatic endothelial cells were cultured in slices and allowed to reach full confluence. Then, the monolayers were stained with a CytoPainter Cell Tracking Staining Kit (Abcam) and inserted into a flow chamber. Calcein AM-labelled (Donjindo) PDAC cells were resuspended (1 × 10^5^ cells per ml) in serum-free culture medium with 0.88 μM fibrinogen and 2.5 mM CaCl_2_ (ref. 21) and loaded into a syringe pump. The dynamic adhesion assays were performed at a shear stress in the range of 0.1–4.0 dynes cm^−2^ according to the manufacturer's manual. The adhered tumour cells on the monolayers were imaged with a fluorescence microscope and the average number of adhered tumour cells in five random fields of × 100 magnification was calculated.

For the static monolayer adhesion assay, Calcein AM-labelled (Donjindo) PDAC cells (1 × 10^5^ cells per well) were added onto the fully confluent monolayers and co-cultured with or without 0.88 μM fibrinogen for 60 min at 37 °C. For the antibody-mediated blocking of cell adhesion, the monolayers were preincubated with 10 μg ml^−1^ anti-human ICAM1 antibody for 1 h at 37 °C.

### Transendothelium migration essay

The HUVECs were seeded in 24-well Transwell inserts with a pore size of 8 μm and grown for 2 days until full confluence. Approximately 200 μl, 5 × 10^5^ ml^−1^ Calcein AM-labelled (Donjindo) tumour cells were added into the apical chamber. The basolateral chambers were filled with 600 μl culture medium (1,640 with 10% FBS), which was used to suspend the tumour cells in the apical chambers. After incubation for 12 h, the apical side of the apical chamber was scraped gently with cotton wool. Only the migrated tumour cells were detected by fluorescence microscopy and counted from five random fields of × 200 magnification.

For the antibody-mediated blocking of TEM, the HUVECs were incubated with 10 μg ml^−1^ anti-human ICAM1 antibody for 1 h at 37 °C. For the fibrinogen-mediated TEM, the Calcein AM-labelled tumour cells were pretreated with 0.88 μM fibrinogen for 2 h at 37 °C.

### Extravasation analysis *in vivo*

The extravasation assays *in vivo* were based on a previously described methodology^48^, with some modifications. Briefly, after anaesthesia, 100 ml of 1 × 10^7^ cells per ml DiI-labelled tumour cells in PBS was injected via the portal vein into green fluorescent protein-knockin mice. Six or 24 h after injection, the abdominal aortas were cut to provide outflow and the livers were perfused with PBS by using gravity pressure through the heart. When the effluent became clear, the livers were frozen in liquid nitrogen, sliced and sealed with a 4,6-diamidino-2-phenylindole-containing mounting medium (Vectorlabs). The sections were immediately imaged by fluorescence microscopy and the tumour cells within the hepatic sinusoid or extravasated into the liver tissue were counted. The total number of adhered or extravasated tumour cells in 20 equally spaced sections of each mouse was calculated. Each group contained three mice. The average numbers per 10 mm^2^ were determined. All of the experimental protocols and animal care were approved by the Ethics Committee of the Tianjin Medical University Cancer Institute and Hospital, and were in compliance with the principles and procedures outlined in the NIH Guide for the Care and Use of Laboratory Animals.

### Orthotopic tumour model

Four weeks old female SCID mice were maintained in a barrier facility on high-efficiency particulate air-filtered racks. All animal studies were approved by the Ethics Committee of the Tianjin Medical University Cancer Institute and Hospital and conducted by skilled experimenters under an approved protocol in accordance with the principles and procedures outlined in the NIH Guide for the Care and Use of Laboratory Animals. Tumour cells were harvested by trypsinization, washed in PBS and resuspended at 1 × 10^7^ cells per ml in Matrigel. The peritoneal cavities of the mice were opened and a total of 1 × 10^6^ cells were transplanted into the pancreases of nude mice. Six weeks later, the numbers of visible metastases in the gut, mesentery and liver, as well as invisible micrometastases, were calculated. Six sets of mice were used in this assay, each with eight mice.

### TCGA data analysis

The TCGA Data of 187 patients with pancreatic carcinoma were downloaded from the website of TCGA. Among them, 183 patients had complete RNA sequencing data. Twenty-six patients were excluded for not having pancreatic adenocarcinoma according to the annotations. The normalized counts of the RNA expression of *EBI3*, *P35* and *ICAM1* were extracted and analysed.

### Reverse transcription–PCR

The total RNA of the cells was extracted with TRIzol (Invitrogen) according to the manufacturer's instructions. Then, the mRNA was reversely transcribed to single-stranded complementary DNAs by using a RT–PCR system (TaKaRa). The primers are listed in Supplementary Table 2. Then, real-time fluorescent quantitative PCR or semi-quantitative PCR was used to analyse the cDNA levels. The products of semi-quantitative PCR were detected by agarose gel electrophoresis, and β-actin was used as a loading control.

### Western blotting

Whole-cell extracts were prepared by lysing the cells with RIPA lysis buffer supplemented with a proteinase inhibitor cocktail (Sigma). A total of 30 mg protein lysate was separated by SDS–PAGE and then the target proteins were detected by western blot analysis with the antibodies to EBI3, P35, GP130, IL-12Rβ2, ICAM1, STAT1, STAT4, p-STAT1, p-STAT4 and β-actin (Supplementary Table 1). (The uncropped western blotting scans were presented in Supplementary Fig. 14).

### Cell invasion and migration assay

Cell invasion assays were performed in 24-well plates with 8.0 mm pore inserts pre-coated with Matrigel. For this assay, the tumour cells were digested and resuspended in FBS-free DMEM culture medium. A total of 200 μl (5 × 10^5^ cells per ml) of tumour cells was seeded into the upper chamber. The basolateral chambers were supplemented with 600 μl complete culture medium (DMEM with 10% FBS) and the cells were incubated for 24 h. For each insert, the invading cells in five random fields of × 200 magnification were counted. Each experiment was performed in triplicate and the mean values are presented.

For the migration assays, a total of 5 × 10^4^ tumour cells were seeded into 8.0 mm pore inserts without Matrigel. The migration time was 6 h.

### CCK8 assay

CCK8 assays were performed according to the manufacturer's manual. Briefly, 2,000 cells in 100 μl culture were added into each well of a 96-well plate. The plate was incubated for the indicated time periods (12, 24, 36, 48 and 60 h) in an incubator and 10 μl of CCK-8 solution was added to each well of the plate. The plate was again incubated for 4 h in the incubator and the absorbance at 450 nm was measured using a microplate reader.

### Plasmid construction and stable cell line establishment

The human or mouse fused EBI3-P35 genes were amplified by PCR using commercial IL-35-overexpressing plasmids (InvivoGen) as the templates. Then, the fused IL-35 genes were cloned into pLV-EF1-MCS-IRES-Bsd vectors (Biosettia). Lentiviruses were produced in 293T cells for the stable transfection of the cell lines, per the manufacturer's instructions, and an empty vector was transfected into cells to be used as controls. A total of 1 × 10^5^ tumour cells in 2 ml medium with 8 μg ml^−1^ polybrene were infected with 1 ml lentivirus supernatant. 48 h later, blasticidin (InvivoGen) was added for selection.

For the cell lines with stable knockdown, shRNA sequences were designed with Biosettia's shRNA designer (http://biosettia.com/support/shrna-designer). Three recommended sequences for each of the *ICAM1*, *P35* and *EBI3* genes were synthesized and cloned into the pLV-hU6-EF1α-puro or pLV-mU6-EF1α-puro vectors (Biosettia). Then, the lentiviruses were produced in 293T cells. Scrambled sequences were transfected into the cells to be used as controls. Tumour cells were simultaneously infected with sh-P35 and sh-EBI3 viruses to knock down the IL-35 expression. Of the three stable cell lines, the most efficient one was used for the relevant assays.

### ChIP and ChIP:reChIP assay

ChIP assays were performed using a CHIP kit (Millipore), according to the manufacturer's instructions. Briefly, Panc-1 cells were pretreated with or without IL-35 (100 ng ml^−1^) for 90 min and then immunoprecipitated with anti-STAT1 or anti-STAT4 antibodies. The immunoprecipitated products were detected by RT–PCR analysis. For the CHIP:reCHIP analysis, the Panc-1 cells were first immunoprecipitated with anti-STAT1 antibody and then immunoprecipitated again with anti-STAT4 (S1–S4). The PCR primers are listed in Supplementary Table 2.

### Conventional CD4+ T-cell proliferation assay

Conventional CD4+ T cells were isolated from fresh human peripheral blood of healthy volunteers with a Magnetic Cell Isolation Kit (Miltenyi Biotec), according to the manufacturer's instructions. Then, the cells were activated for 3 days with anti-CD3 and anti-CD28 antibodies in the presence of conditioned medium (50%) or commercial recombinant IL-35 (100 ng ml^−1^). Thereafter, an EDU incorporation kit (Ribobio) was used to detect the proliferation of the treated T cells. Representative immunofluorescence images of the EDU incorporation assay were captured, and the EDU incorporation rates were calculated.

### Statistical analysis

Statistical analyses were performed with the IBM SPSS Statistics Program. Each experiment was performed in triplicate, and the values are presented as the mean±s.d., unless otherwise stated. The variance between the groups was statistically compared. Student's *t*-test was used to compare the mean values. Kaplan–Meier curves were analysed for relevant variables. The log-rank test was used to analyse the differences in survival times among the patient subgroups. The risk factors associated with the prognoses of these patients were evaluated with Cox's proportional hazard regression model. All probability values had a statistical power level of 90%, and a two-sided level of 5%. *P*<0.05 was considered to be significant.

### Data availability

The RNA-sequencing data have been deposited in the GEO database (accession code: GSE85050) and ArrayExpress database (accession code: E-MTAB-3710). The TCGA data referenced in the study are available in a public repository from TCGAwebsite (http://cancergenome.nih.gov/).

The authors declare that all the other data supporting the findings of this study are available within the article and its Supplementary Information files, and from the corresponding author upon reasonable request.

## Additional information

**How to cite this article:** Huang, C. *et al*. Tumour-derived Interleukin 35 promotes pancreatic ductal adenocarcinoma cell extravasation and metastasis by inducing ICAM1 expression. *Nat. Commun*. **8**, 14035 doi: 10.1038/ncomms14035 (2017).

**Publisher's note:** Springer Nature remains neutral with regard to jurisdictional claims in published maps and institutional affiliations.

## Supplementary Material

Supplementary Information Supplementary Figures and Supplementary Tables

## Figures and Tables

**Table 1:** Association between IL-35 expression and clinicopathological features of patients with pancreatic carcinoma tissues.

	Total	IL-35 expression		*χ*^2^	*P*-value
		High	Low/absent		
Gender				0.982	0.210
Male	72	33	39		
Female	51	28	23		
Age (years)				0.492	0.315
<65	96	46	50		
≥65	27	15	12		
Differentiation				6.610	0.009*
Good/moderate	93	40	53		
Poor	30	21	9		
Pathological TNM				8.648	0.003*
I	35	10	25		
II–III	88	51	37		
Nodal involvement				4.447	0.028*
−	91	40	51		
+	32	21	11		
Blood vessel infiltration				8.602	0.003*
−	82	33	49		
+	41	28	13		
Tumour size (cm)				3.443	0.049*
<5	88	39	49		
≥5	35	22	13		

IL-35, interleukin-35.

*P<0.05 (*χ*^2^-tests); exact significance (two-sided).

**Table 2:** Cox's proportional hazard model analysis of prognostic factors in patients with pancreatic carcinoma.

Variables	Unfavourable/favourable	Overall survival		Relapse-free survival	
		HR (95% CI)	*P*-value	HR (95% CI)	*P*-value
*Univariate analysis*					
IL-35	High/low or absent	2.160 (1.465–3.186)	0.0001*	1.985 (1.280–3.079)	0.0020*
Sex	Female/male	0.968 (0.668–1.402)	0.8610	1.048 (0.692–1.586)	0.8260
Age	≥65/<65	0.978 (0.631–1.516)	0.9780	0.747 (0.450–1.239)	0.2580
Differentiation	Poor/good or moderate	2.264 (1.478–3.468)	0.0002*	3.581 (2.257–5.681)	<0.0001*
Tumour size	≥5/<5	2.841 (1.827–4.419)	<0.0001*	2.514 (1.545–4.091)	0.0002*
Nodal involvement	+/−	2.408 (1.590–3.646)	<0.0001*	2.098 (1.280–3.440)	0.0030*
Vessel infiltration	+/−	2.939 (1.968–4.389)	<0.0001*	3.479 (2.210–5.478)	<0.0001*

*Multivariate analysis*					
IL-35	High/low or absent	1.765 (1.142–2.730)	0.0110*	—	0.0710
Differentiation	Poor/good or moderate	2.178 (1.382–3.432)	0.0010*	3.442 (2.105–5.630)	<0.0001
Tumour size	≥5/<5	2.671 (1.680–4.245)	<0.0001*	2.369 (1.147–3.234)	0.0130*
Nodal involvement	+/−	1.854 (1.182–2.908)	0.0070*	1.926 (1.147–3.234)	0.0130*
Vessel invasion	+/−	1.770 (1.105–2.883)	0.0170*	2.432 (1.476–4.009)	0.0005*

CI, confidence interval; HR, hazard ratio; IL-35, interleukin-35.

Multivariate Cox's analysis method: forward: LR; *, *P*<0.05; exact significance (2-sided).
