# Supplementary material for: Tumour-derived Interleukin 35 promotes pancreatic ductal adenocarcinoma cell extravasation and metastasis by inducing ICAM1 expression
Source: Nat Commun. 2017 Jan 19;8:14035. doi: 10.1038/ncomms14035 (PMC5253665; doi:10.1038/ncomms14035)
Supplement: Supplementary Information — Supplementary Figures and Supplementary Tables [file ncomms14035-s1.pdf]

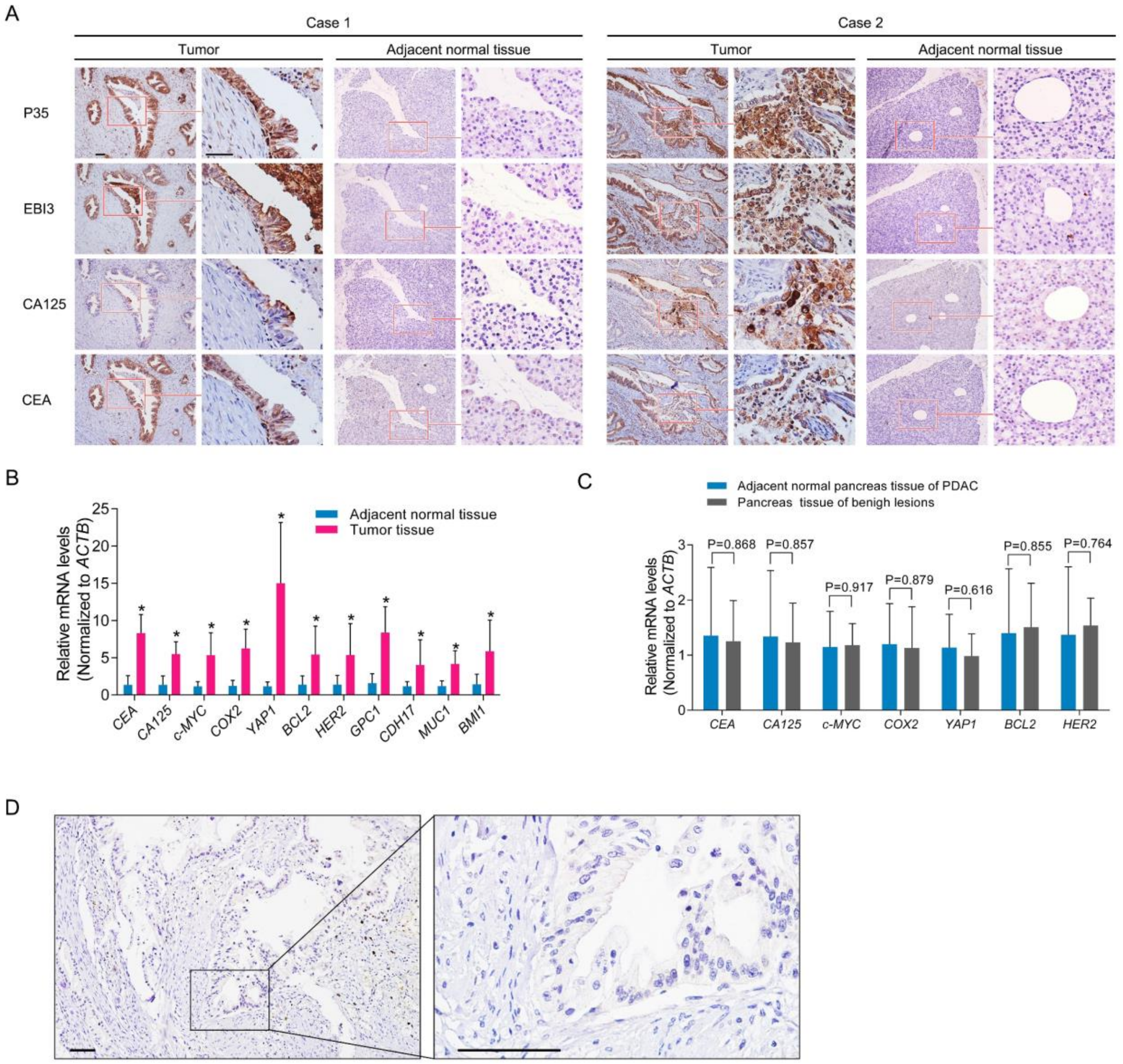

Supplementary Figure 1. The expression levels of cancer-related genes in PDAC tissues and the corresponding adjacent normal tissues. A, immunohistochemistry staining for EBI3, P35, CA125 and CEA in consecutive sections of pancreatic carcinoma tissues and the adjacent normal pancreatic tissues (2-3 cm around the tumor border). B, the mRNA expression levels of 11 cancer-related genes detected by RT-PCR in 10 PDAC tissues and the corresponding adjacent normal tissues. C, the mRNA levels of *CEA, CA125, c-MYC, COX2, YAP1, BCL2* and *HER2* in the pancreas tissue of patients with PDAC and duodenal carcinoma (used as normal controls). D, immunohistochemical staining with no primary antibodies was shown as a negative control. Unpaired t test was used; shown are mean ± S.D. Experiments were replicated for three times. Tests of significance are two-sided. *$P < 0.05$. Figure panels pairs in A and D represent images taken with different zooming options; scale bars, 100μm.

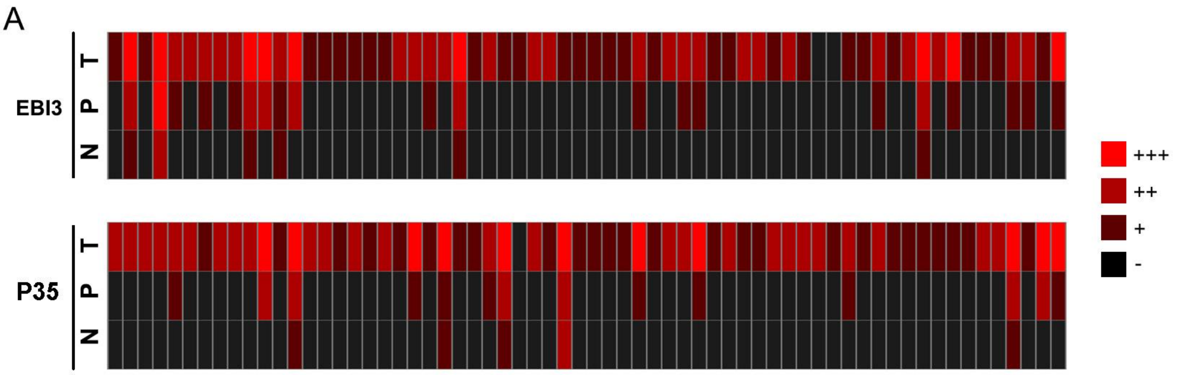

B

	Tumor vs. Pre.	Pre. vs Normal	Tumor vs normal
EBI3	Z = -7.168<br>P < 0.0001	Z = -4.185<br>P < 0.0001	Z = -7.025<br>P < 0.0001
P35	Z = -7.130<br>P < 0.0001	Z = -3.276<br>P = 0.001	Z = -7.064<br>P < 0.0001

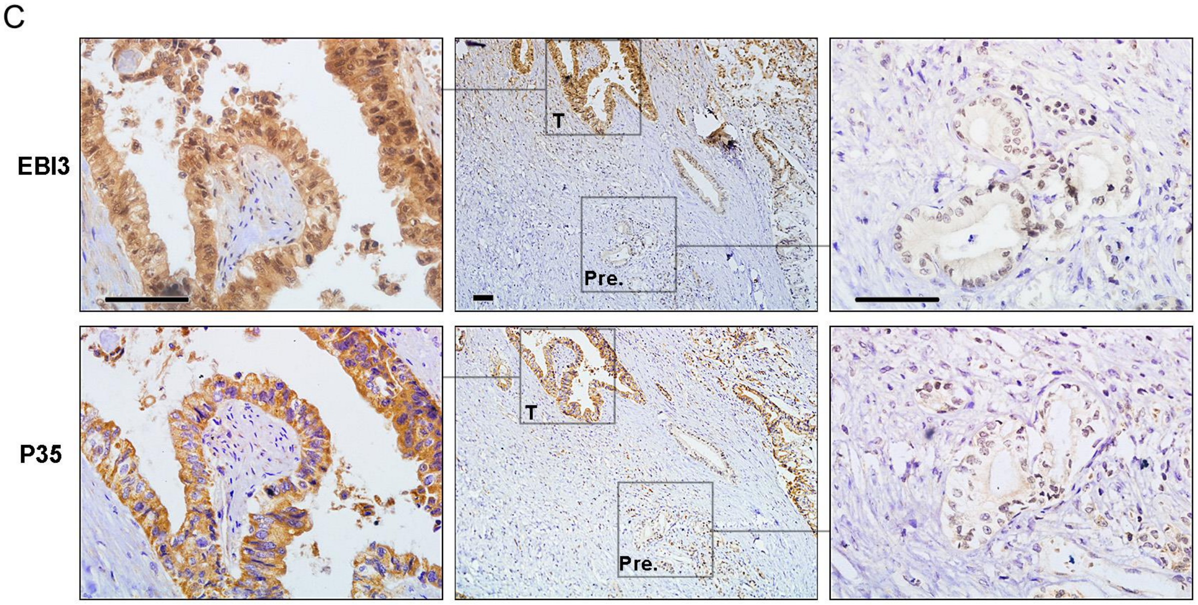

Supplementary Figure 2. The expression of IL-35 subunits in PDAC tissues, adjacent pre-neoplastic lesions and adjacent normal pancreas tissues. A, the heat map showing immunohistochemical staining of EBI3 and P35 in PDAC tumor tissues, the corresponding adjacent pre-neoplastic lesions and corresponding adjacent normal pancreas tissues; N=64. B, Wilcoxon Test was used to analyze the differential expression of EBI3 and P35. C, representative images of differential IL-35 expression in pancreatic ductal adenocarcinoma tumor tissue and the adjacent pre-neoplastic tissue. T, tumor; P/Pre., pre-neoplastic lesions; N, normal pancreas tissues. Tests of significance are two-sided. *$P < 0.05$. Figure panels pairs in C represent images taken with different zooming options; scale bars, 100μm.

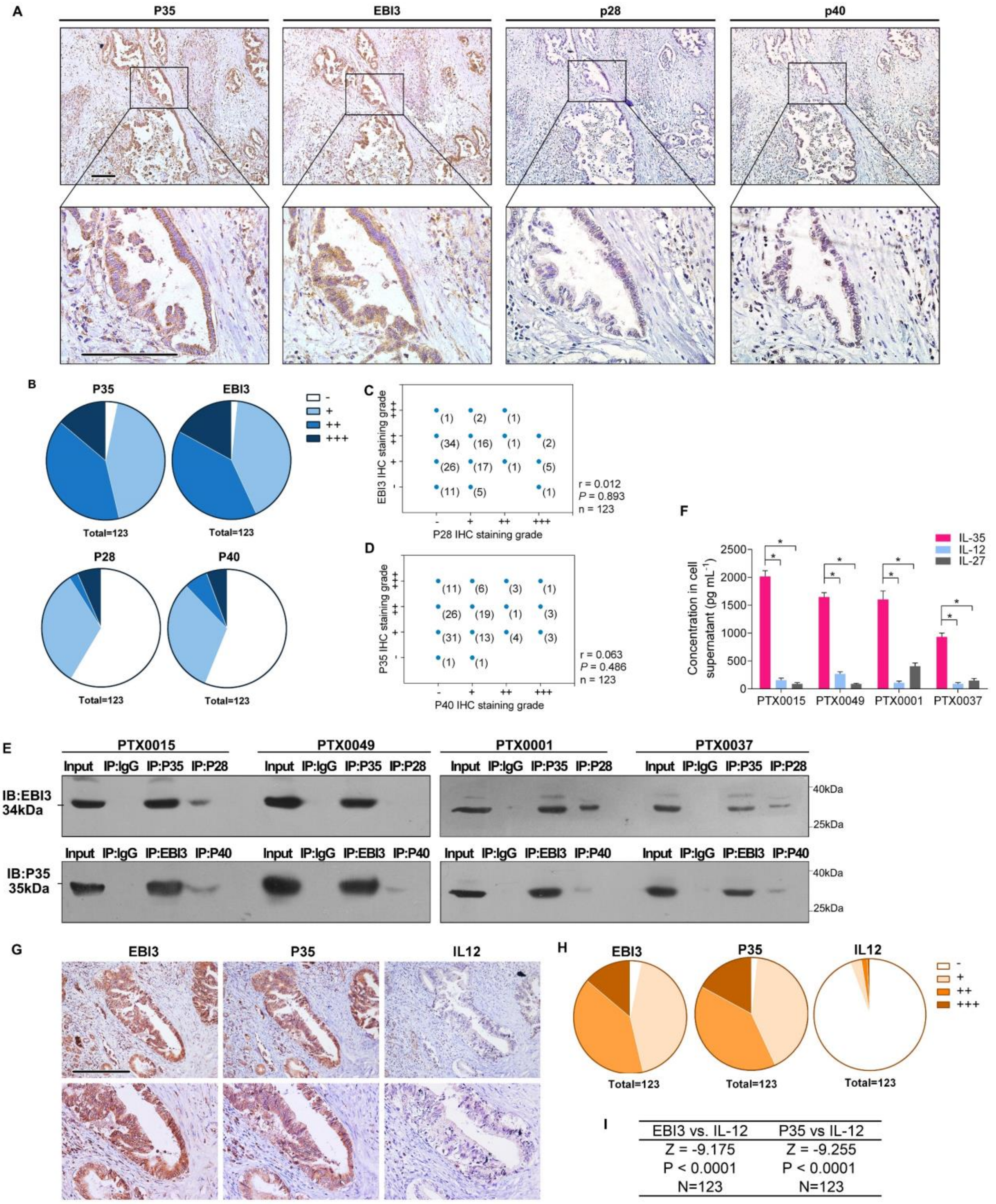

Supplementary Figure 3. The correlation between EBI3, P35, P28 and P40 expression in specimens of PDAC patients. A, immunohistochemical analysis of EBI3, P35, P28 and P40 correlative expression in consecutive sections from human PDAC surgical samples. Scale bars, 200 μm. B, the real distribution of immunohistochemical results of EBI3, P35, P28 and P40. C-D, statistical analysis of immunohistochemical results of EBI3 and P28 expression (C), as well as that of P35 and P40 (D) in human PDAC surgical samples. E, Co-immunoprecipitation and western blot

analysis of EBI3:P35, EBI3:P28 and P35:P40 protein from lysates of 4 primary PDAC cell lines. F, a total of 5 × 10^5 cells were seeded onto flask and cultured in 4 mL medium for 24 hours. ELISA assays were done to detect the levels of IL-35, IL-12 and IL-27 in supernatant of 4 primary PDAC cell lines. G, immunohistochemical analysis of EBI3, P35 and IL-12(IL-12p70) expression in consecutive sections from human PDAC surgical samples. H, the real distribution of immunohistochemical results of EBI3, P35 and IL-12. I, Wilcoxon Test used to analyze the differential IL-12 levels. Figure panels pairs in A and G represent images taken with different zooming options; scale bars, 100 μm.

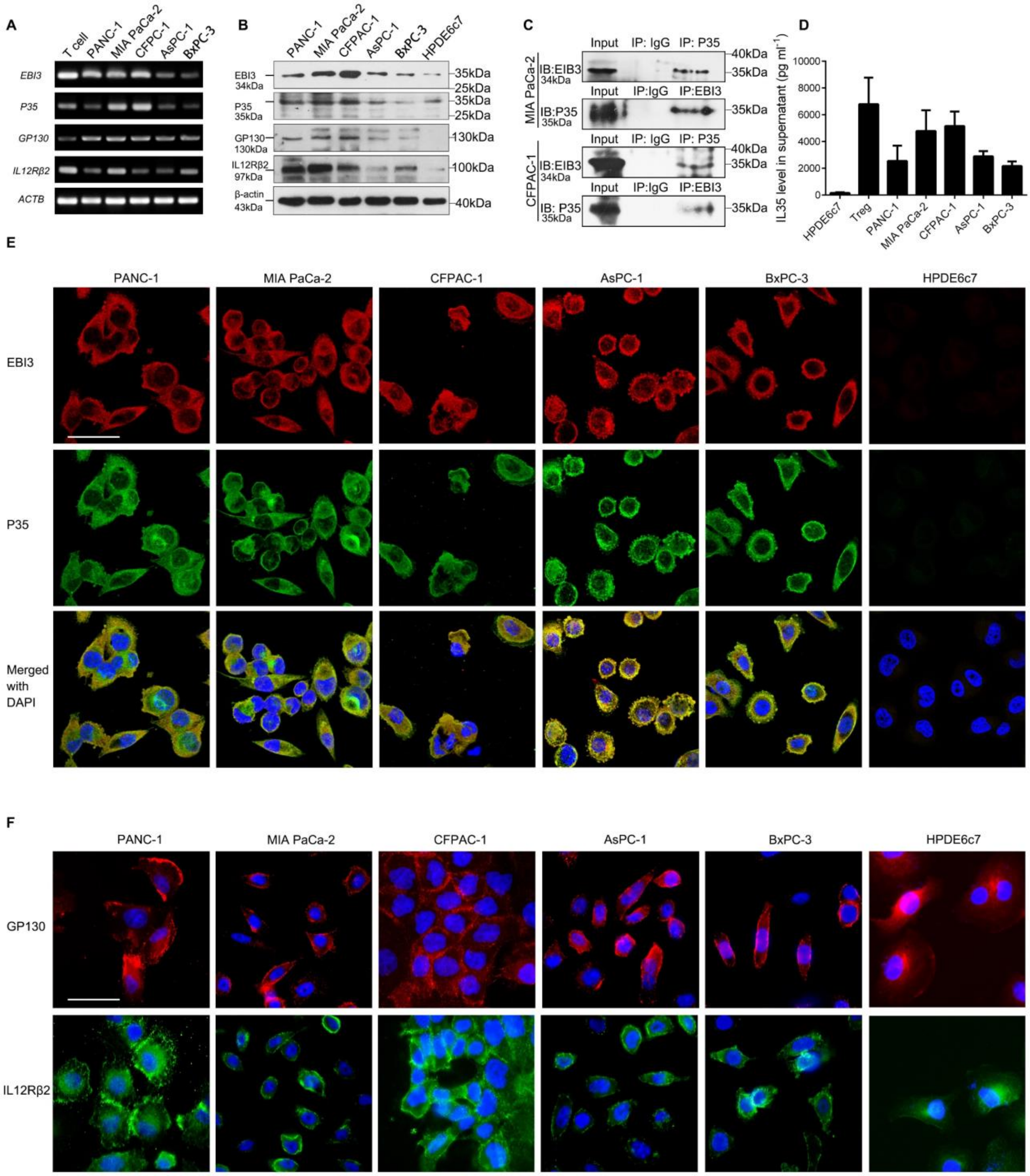

Supplementary Figure 4. The expression of IL-35 ligand and receptor in PDAC cell lines. A-B, expression levels of the two subunits of IL-35 (EBI3 and p35) and the two subunits of IL-35 receptor (gp130 and IL12rβ2) in five PDAC cell lines (Panc-1, BxPC-3, AsPC-1, MIA PaCa-2 and CFPAC-1) and one normal pancreatic ductal epithelial cell line (HPDE6c7) were detected by RT-PCR (A) and Western blot (B). CD4+CD25+ Treg (nTreg) cells were used as positive expression control; β-actin was used as a loading control. C, co-immunoprecipitation and western blot analysis of EBI3 and p35 protein from lysates of MIA PaCa-2 and CFPAC-1 cells. D, a total of 5 × 10^5 cells were cultured in 4

mL medium for 24 hours. The supernatant was subjected to ELISA assays to detect IL-35 levels. Unpaired t test was used. Tests of significance are two-sided. *$P < 0.05$. E, subcellular localization of endogenous EBI3 and p35 in 5 PDAC cell lines. EBI3 and p35 was co-located in the cytoplasm of the cells. Immunofluorescence images were taken by a confocal microscopy. Scale bars, 50μm. F, the membrane localization of gp130 and IL12rβ2 was detected by a microscopy after immunofluorescent staining. Scale bars in E and F, 100μm.

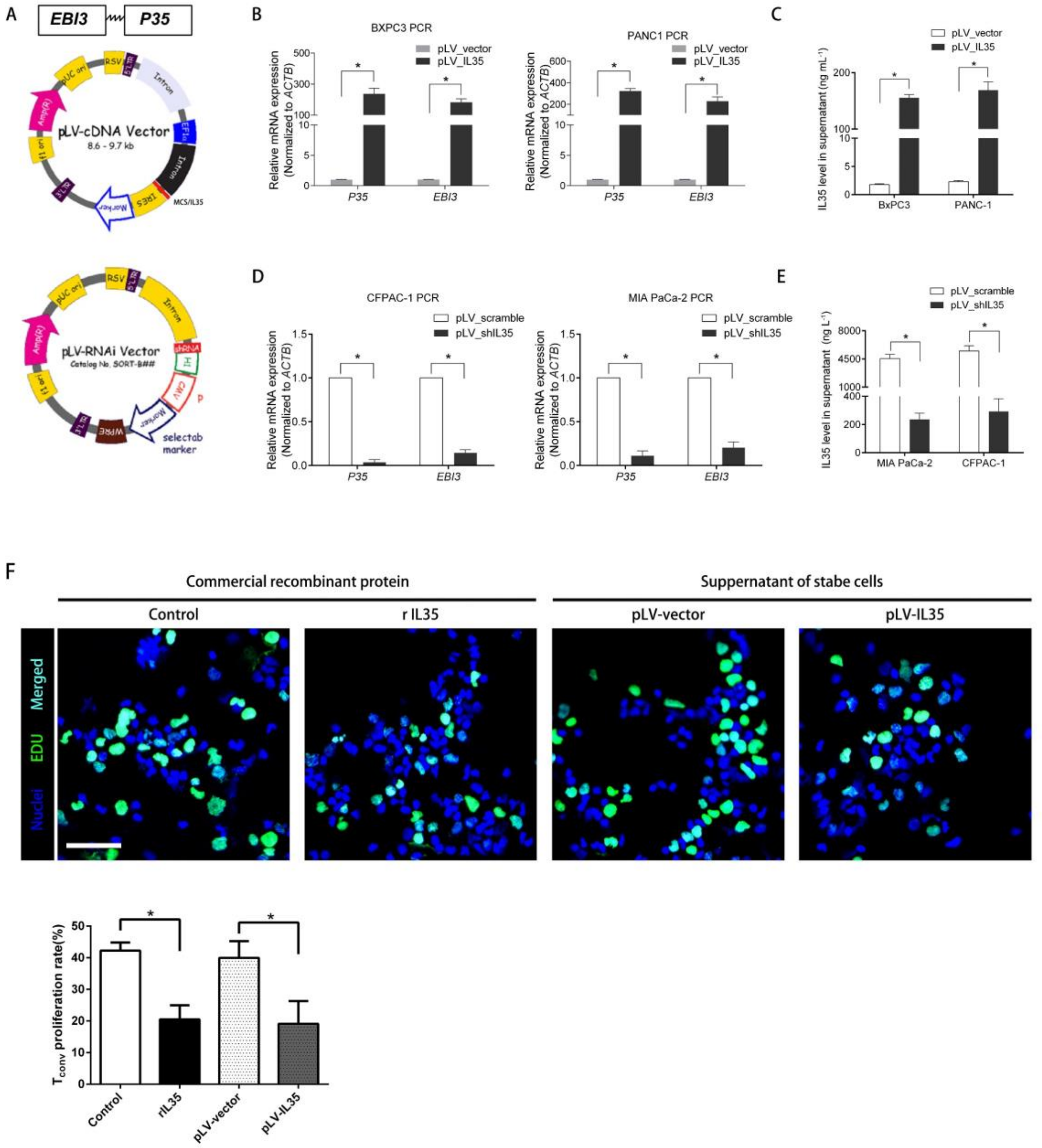

Supplementary Figure 5. Establishment and verification of stable cells lines overexpressing or downexpressing IL-35. A, the schematic of construction of IL-35-overexpressing and IL-35-downexpressing cell lines is presented; the EBI3-P35 fusion sequence was cloned from a commercial IL-35-expressing plasmid (InvivoGen). B-C, PANC-1 and BxPC-3 cells were stably transfected with the fused EBI3-p35 gene, while empty vectors were transfected as controls. RT-PCR (B) and Elisa analysis (C) were used to detect the production and secretory level of IL-35. D-E, MIA PaCA-2 and CFPAC-1 cells were stably transfected with both shEBI3 and shP35 sequences to knockdown IL-35 expression, while scrambled sequences were transfected as controls. RT-PCR (D) and Elisa analysis (E) were used to detect the production and secretory level of IL-35. F, verification the biological function of fused IL-35 protein and commercial recombinant IL-35. Human conventional $CD4^+$ T cells were purified by a magnetic beads sorting kit from peripheral blood of healthy volunteers, then activated for 3 days with anti-CD3 and anti-CD28 antibodies in the presence of conditioned medium from the IL-35-overexpressing PANC-1 cells (50%) or commercial recombinant IL-35

(100ng/mL). Thereafter an EDU incorporation kit was used to detect the proliferation of the cells. Representative immunofluorescence images of the EDU incorporation assay (top). Scale bars, 50 μm. Nuclei of T cells were labelled with blue color (Hoechst33342) and EDU were labelled with green color (Apollo®488). Statistical results are presented as the average EDU incorporation rates of 5 randomed fields of 400 × magnifications. Data represent three independent experiments (mean±S.D.) (bottom). Experiments were replicated for three times. Unpaired t test was used; Shown are mean values ± SD; *P < 0.05; tests are two-sided.

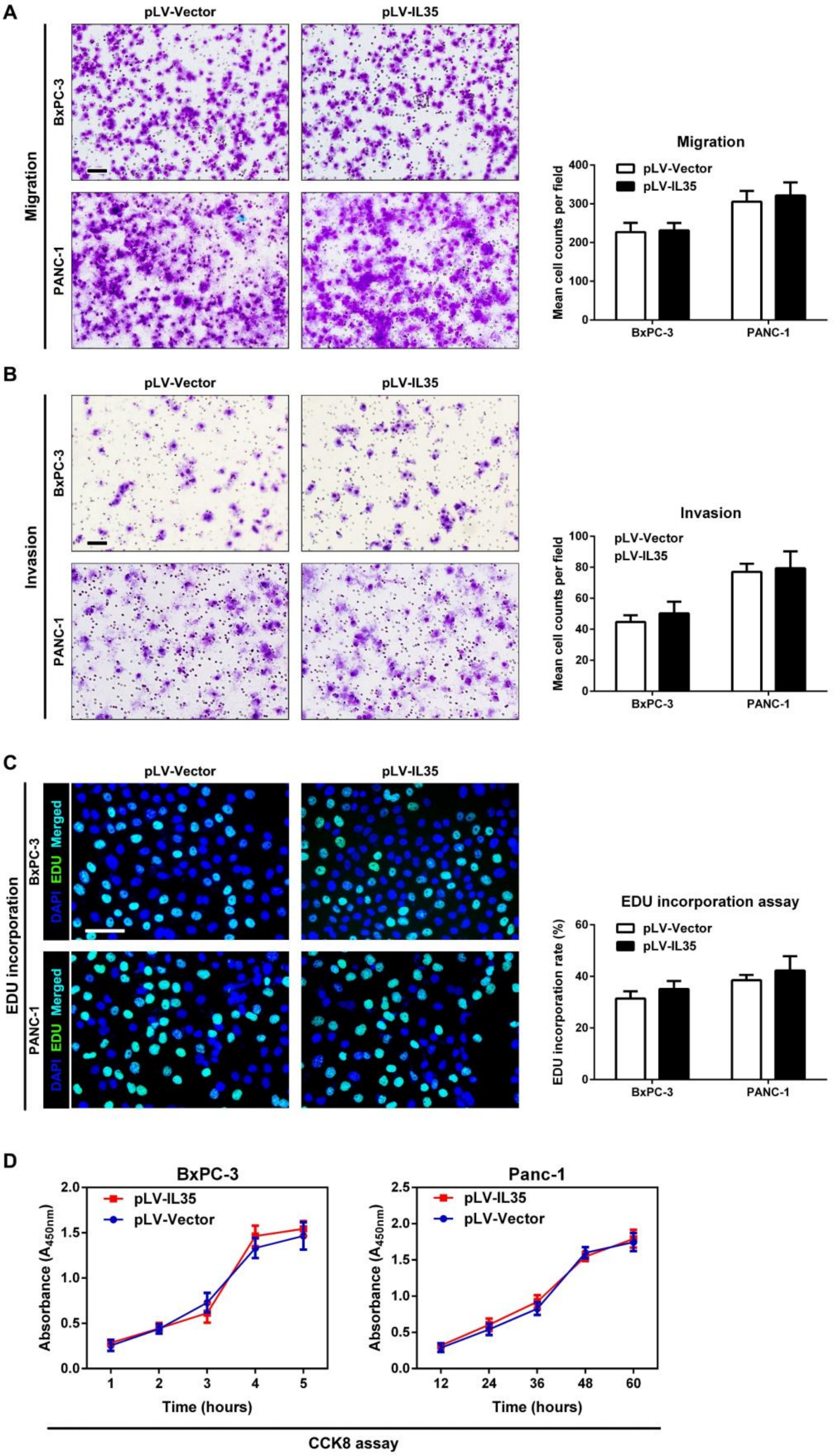

Supplementary Figure 6. The roles of IL-35 in PDAC migration, invasion and proliferation

assays. Panc-1 and BxPC-3 cells stably transfected with pLV-IL35 or pLV-Vector were subjected to Transwell migration assays (A), Transwell invasion assays (B), EDU incorporation assays (C) and the CCK8 assays (D). The experiments were performed in triplicate. Experiments were replicated for three times. Unpaired t test was used; Shown are mean values ± SD; **P* < 0.05; tests are two-sided. Scale bars, 100 μm.

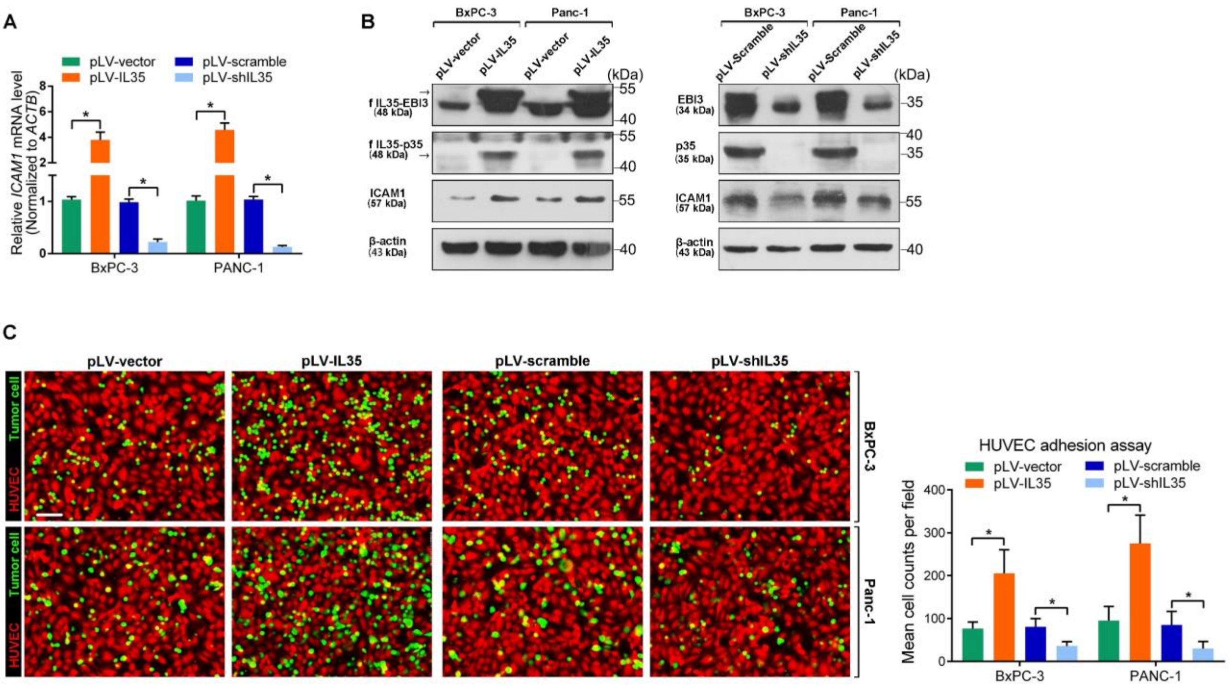

Supplementary Figure 7. IL-35 facilitates PDAC cells adhesion to endothelial monolayer in static adhesion assays. Stable cell lines of BxPC-3 and PANC1 with IL-35 down-regulated or up-regulated were detected by RT-PCR (A) and Western blot assays (B). Then the stable cell lines were subjected to static HUVEC adhesion assays (C). The detailed procedure was shown in Method. Experiments were replicated for three times. Unpaired t test was used; Shown are mean values ± SD; *$P < 0.05$; tests are two-sided. Scale bars, 100 μm.

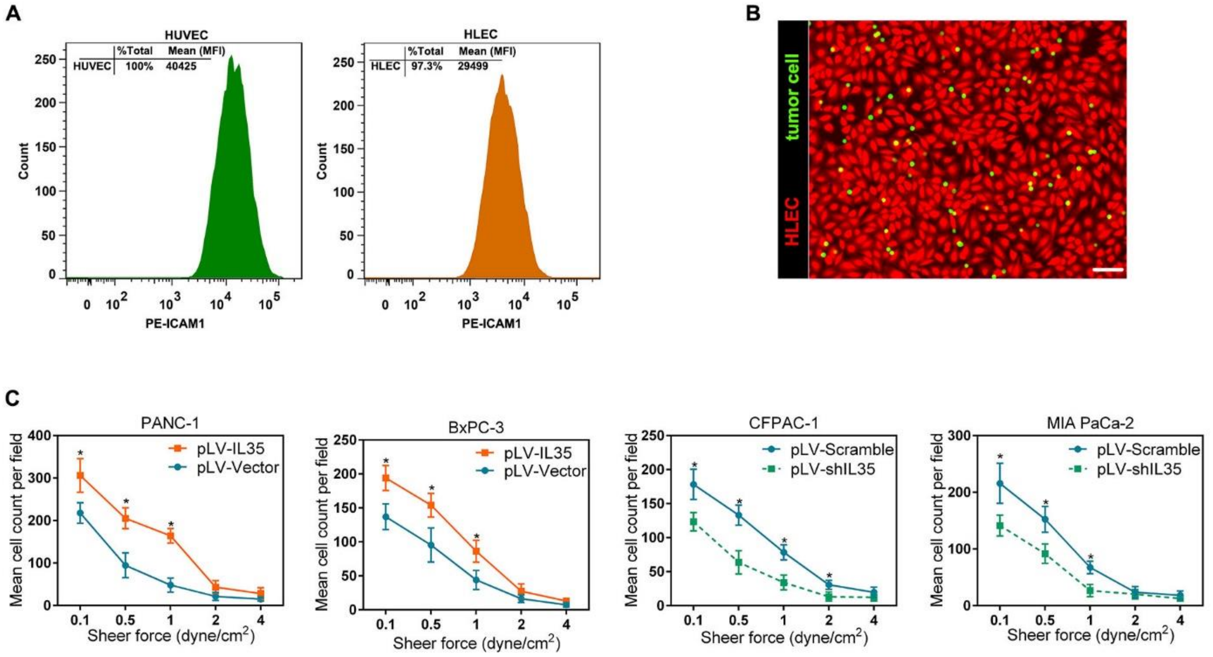

Supplementary Figure 8. IL-35 enhances the PDAC - human lymphatic endothelial cells (HELC) adhesion in dynamic flow assays. A, the FACS assays was performed to compare ICAM1 expression between HUVECs and HLECs. B, representative immunofluorescence image demonstrates the dynamic flow assays at the shear force of 1 dyne/cm2; scale bar, 100μm. C, the adhesion-shear force curves of the adhesion assays done at shear forces from 0.1 to 4 dyne cm^{-2}. Experiments were replicated for three times. Unpaired t test was used; Shown are mean values ± SD; *P < 0.05; tests are two-sided. Scale bars, 100 μm.

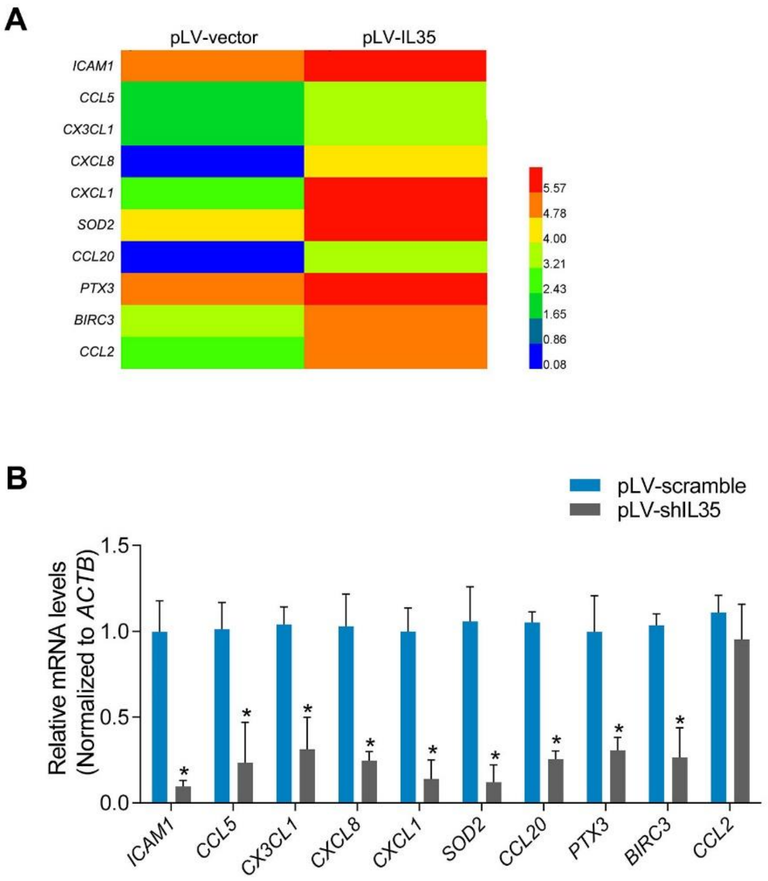

Supplementary Figure 9. Verification of the mRNA sequencing data. A, the mRNA heat-map of 10 genes up-regulated in PANC-1 cells overexpressing IL-35; the normalized counts (RPKM) were normalized to log2 rank. B, RT-PCR assays were perormed to verify the mRNA sequencing results. Experiments were replicated for three times. Unpaired t test was used; Shown are mean values ± SD; **P* < 0.05; tests are two-sided.

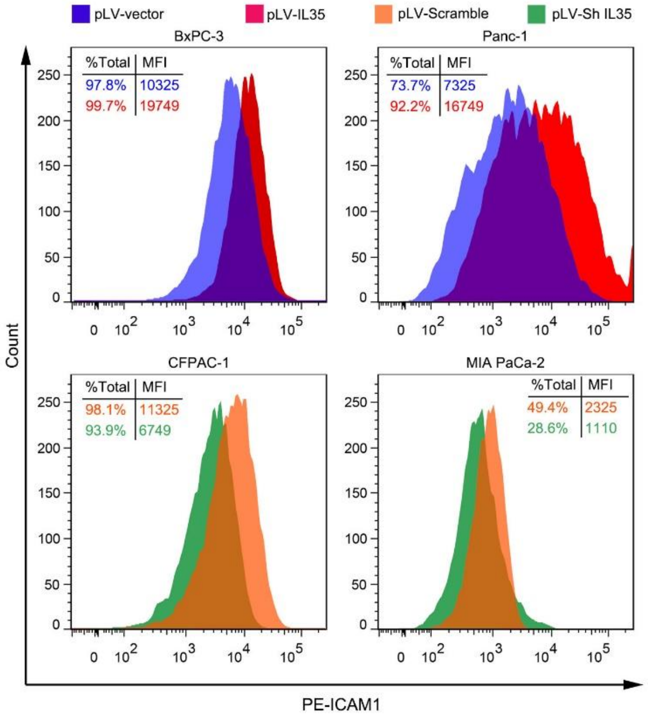

Supplementary Figure 10. The regulating effect of IL-35 to ICAM1 examined by FACS analysis. PDAC cells were stably up-regulated or down-regulated of IL-35 and then subjected to FACS analysis**.**

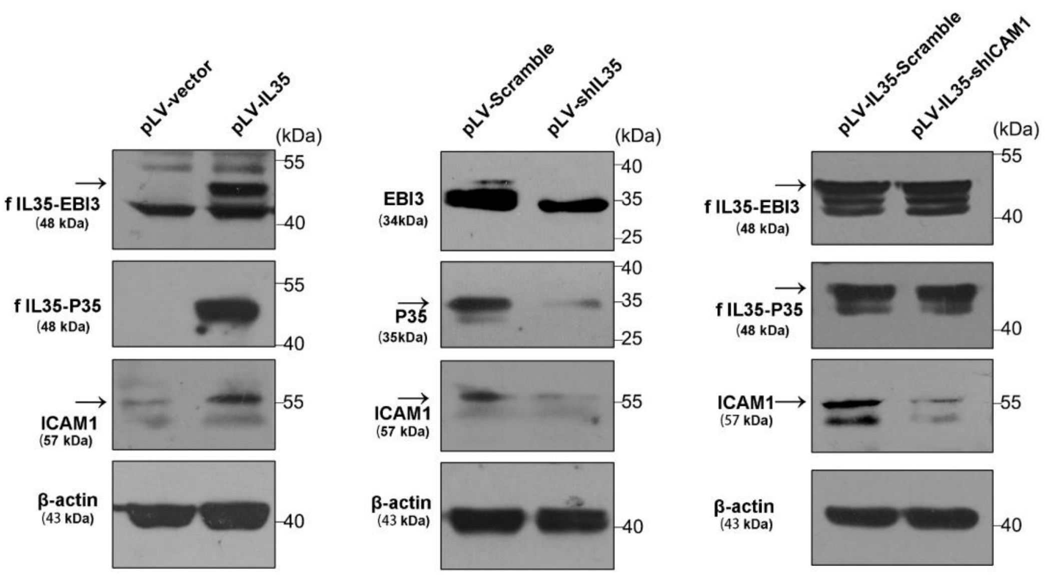

Supplementary Figure 11. The verification of Pan02 stable cell lines. Pan02 stable cell lines were constructed as indicated and subjected to Western blotting assays.

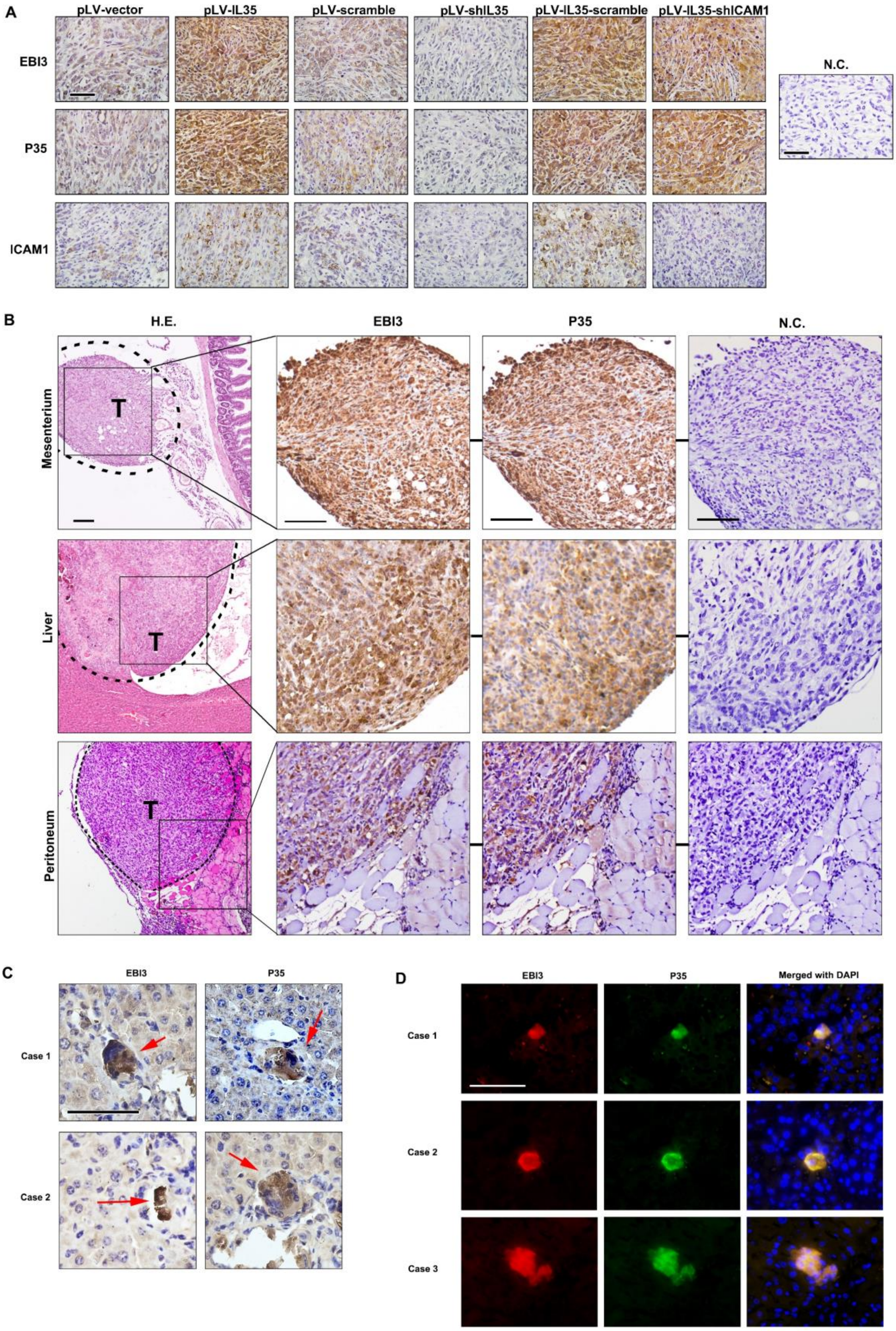

Supplementary Figure 12. SCID mouse model of orthotopically transplanted Pan02 tumor cells. A, six weeks after the Pan02 cells were orthotopically transplanted, the tumors and metastases

were harvested and subjected to immunohistochemistry staining to detect the expression level IL-35 (EBI3 and p35) and ICAM1. An Immunohistochemical staining with no primary antibodies was shown as the negative control (N.C.). B, representative H.E. staining and immunohistochemistry staining of visible metastases in peritoneum, mesenterium and liver. Immunohistochemical stainings without primary antibodies was used as the negative controls (N.C.). C, representative immunohistochemistry staining for EBI3 and P35 in consecutive sections of invisible micrometastases in mice liver. D, immunofluorescence staining for the co-locolization of EBI3 and P35 in invisible micrometastases in mice liver. Red arrows, micro-metastases; Figure panels pairs represent images taken with different zooming options; scale bars, 100μm.

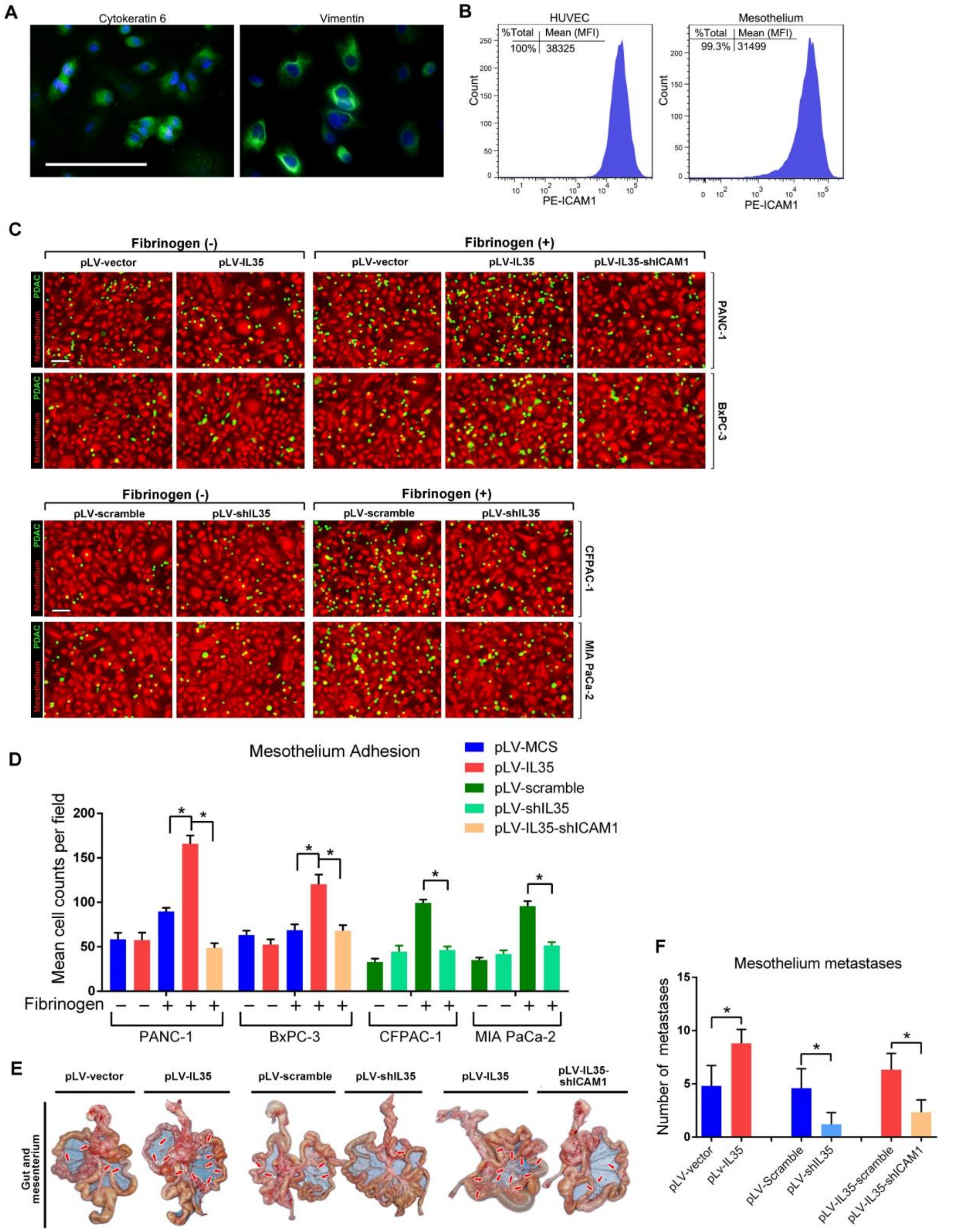

Supplementary Figure 13. IL-35-ICAM1 axis promotes PDAC adhesion and metastasis to mesothelium. A, the primary mesothelial cells was extracted from human epiploon donated by PDAC patients, then the mesothelial cells was verified by the staining of mesothlial markers: cytokeratin and Vimentin. B, the FACS assays was performed to compare ICAM1 expression between HUVEC and mesothelial cells. C-D, the indicated tumor cells were added onto HUVEC

monolayers and the adhered tumor cells were determined in the absence or presence of fibrinogen. E-F, Representative images (E) and total numbers (F) of metastasis in gut and mesenterium (n = 8 for each group). Unpaired t test was used; Shown are mean values ± SD; *$P < 0.05$; tests are two-sided. Scale bars in A and C, 100 μm.

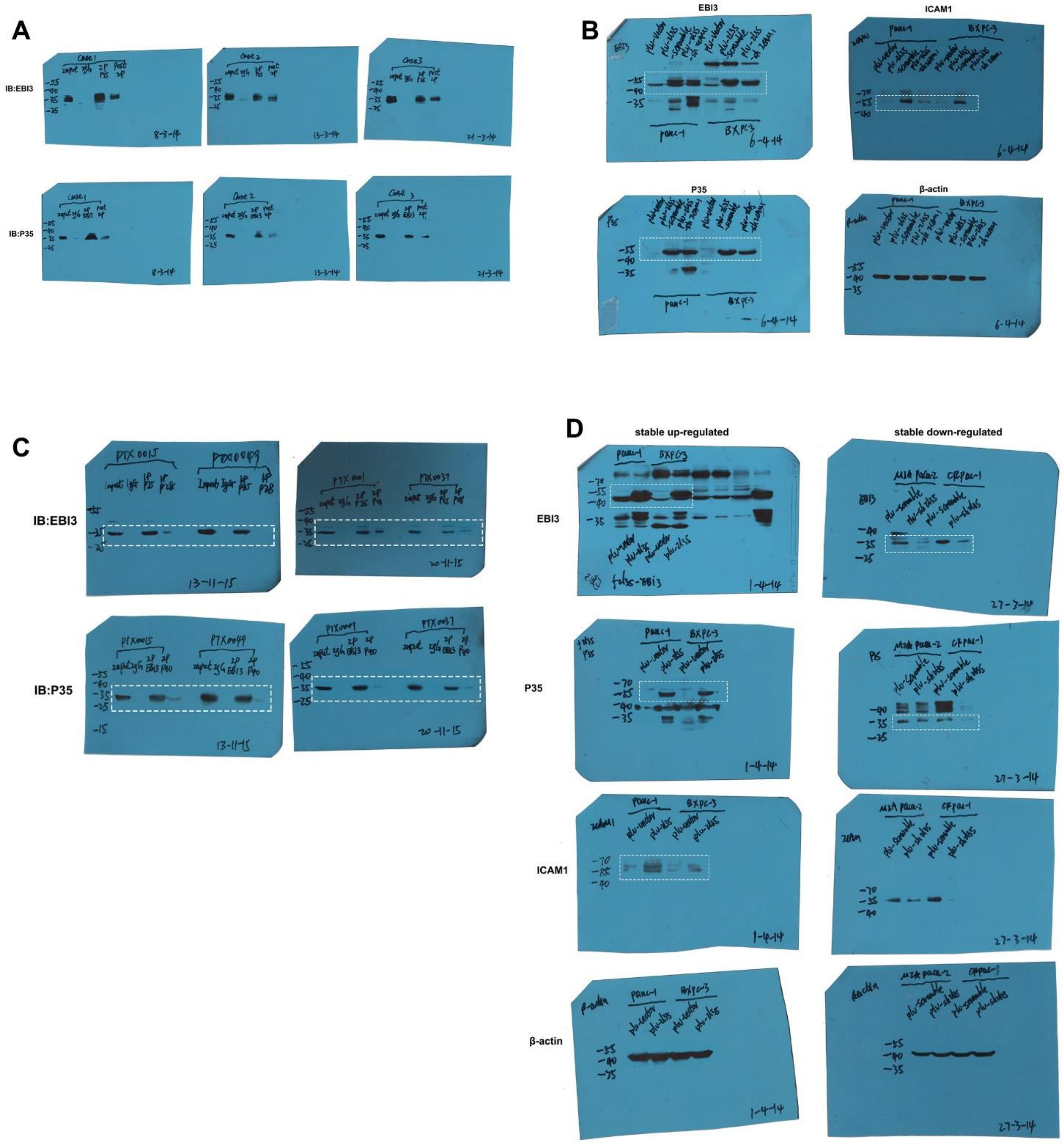

Supplementary Figure 14. Uncropped Western blotting scans. A, Uncropped Western blotting scans for Figure 1G. B, Uncropped Western blotting scans for Figure 3E. C, Uncropped Western blotting scans for Supplementary Figure 3E. D, Uncropped Western blotting scans for 3D.

Supplementary Table 1. Main antibodies and reagents

	Name	Company	Cat.No.	Concentration
**For IHC and WB**	anti-EBI3 antibody	abcam	ab124694	1:10000 (WB)
	anti-EBI3 antibody	abcam	ab83896	1:400 (IHC)
	anti-gp130 antibody	abcam	ab34315	1:2000 (WB)
	anti-gp130 antibody	SANTA CRUZ	sc-9045	1:200 (IHC)
	anti-IL12RB2 antibody	abcam	ab96097	1:1000 (WB)
	anti-IL12RB2 antibody	LifeSpan	LS-C165173	1:100 (IHC)
	anti-IL12A p35 antibody	abcam	ab136323	1:500 (WB)
	anti-IL12A p35 antibody	abcam	ab131039	1:250 (IHC)
	anti-ICAM-1 antibody	Leica	NCL-CD54-307	1:50 (IHC)
	Anti-ICAM1 antibody	abcam	ab171123	1:1000 (WB)
	anti-Stat1 antibody	CST	#9172	1:1000 (WB)
	anti-Stat4 antibody	CST	#2653	1:1000 (WB)
	anti-P-Stat1 antibody	CST	#9167	1:1000 (WB)
	anti-P-Stat4 antibody	CST	#5267	1:1000 (WB)
	anti-IL12 p40 antibody	abcam	ab106270	1:200 (IHC)
	anti-IL27 p28 antibody	abcam	ab115671	1:200 (IHC)
	anti-CD34 antibody	abcam	ab81289	1:100 (IHC, IF)
	anti-β-actin antibody	Ray Biotech	RM2001	1:5000 (IHC)
**Neutralizing antibodies**	anti-IL12RB2 antibody	R&D Systems	AF1959	2ug mL-1
	anti-ICAM-1 antibody	R&D Systems	BBA3	10ug mL-1
	anti-gp130 antibody	R&D Systems	MAB288	100ng mL^{-1}
**Fluorescent secondary**	Alexa Fluor488 goat anti-rabbit	Lifetechnologies	A11070	1:800
	Alexa Fluor594 donkey anti-mouse	Lifetechnologies	A21203	1:800
	Alexa Fluor647 goat anti-mouse	Lifetechnologies	A21236	1:800
	Alexa Fluor546 goat anti-rabbit	Lifetechnologies	A11010	1:800
**Other reagents**	Recombinant Human IL35	Sino Biological lnc.	10705-H02H	
	CHIP Kit	Millipore	2506474	
	Human IL-35 ELISA Kit	BioLegend	439508	
	Fibrinogen	Sigma	F3879	
	stat1 inhibitor(Fludarabine)	Selleckchem	S1491	
	Calcein-AM	Dojindo Laboratories	C326	
	CM-DIL	Invitrogen	C7000	
	Collagenase type 2	Gibco	17101-015	
	Endothelial Cell Growth Medium	Lonza	CC-3162	
	CytoPainter Cell Tracking Staining kit	abcam	ab138892	
	pORF9-hIL35elasti	InvivoGEN	porf-hil35	
	pUNO1-mIL35elasti	InvivoGEN	puno1-mil35	
**Co-immunoprecipitation and blotting assay in Fig. 1G and Suppl. Fig. 3E.**	anti-IL27 P28 antibody (Host:Rabbit)	LifeSpan	LS-C310763	IP, 1:20; IB,
	anti-IL-12 P40 antibody (Host:Mouse)	Biolegend	508803	IP, 1:20; IB,
	anti-EBI3 antibody (Host:Mouse)	SANTA CRUZ	sc-166158	IP, 1:20; IB,
	anti-IL12A P35 antibody (Host:Rabbit)	SANTA CRUZ	sc-7925	IP, 1:20; IB, 1:500

Supplementary Table 2. Primers and oligonuceotides sequences.

**Interfering oligonucleotides**	
sh-Human ICAM1	AAAACCGAGCTCAAGTGTCTAAATTGGATCCAATTTAGACACTTGAGCTCGG
sh-Human IL12A	AAAAGAGACCTCTTTCATAACTATTGGATCCAATAGTTATGAAAGAGGTCTC
sh-Human EBI3	AAAATCACGGATGTCCAGCTGTTTTGGATCCAAAACAGCTGGACATCCGTGA
sh-mouse ICAM1	AAAAGCTGGCATTGTTCTCTAATTTGGATCCAAATTAGAGAACAATGCCAGC
sh-mouse IL12A	AAAATGTCCAAGCTGCTCTTCCTGTCACTTGCCTTGGATCCAAGGCAAGTGA CAGGAAGAGCAGCTTGGACA
sh-mouse EBI3	AAAAGGTCCAGCATGTGTCAATCACGCTACCTCTTGGATCCAAGAGGTAGCG TGATTGACACATGCTGGACC
**PCR primers**	
human IL12 P35	
Forward primer	CTCCCTTGAAGAACCGGAT
Reverse primer	ATCAATAGTCACTGCCCGAA
human EBI3	
Forward primer	GATCCGTTACAAGCGTCAGG
Reverse primer	CTCAGTTCCCCGTAGTCT
human IL12rβ2	
Forward primer	AGGAATCCAAGGTCATCAGG
Reverse primer	TCCACCAGTTGTCTGCTCTC
human GP130	
Forward primer	GGAGTGCTGTTCTGCTTTAA
Reverse primer	ACTGTGTACCACGGTAGAAT
human ICAM1	
Forward primer	GAACCCATTGCCCGAGCTCA
Reverse primer	TGACAGTCACTGATTCCCCGAT
human β-actin	
Forward primer	CTACCTTCAACTCCATCATGAAGTG
Reverse primer	TGCGCTCAGGAGGAGC
**CHIP primers**	
ICAM1 binding motif1 primer	
Forward primer	CTTCGTCACTCCCACGGTT
Reverse primer	GTCACGTCCACACCTAGCTGA
ICAM1 binding motif2 primer	
Forward primer	CCGCCCGATTGCTTTAGCTTG
Reverse primer	ATCCTTTATAGCGCTAGCCAC

Supplementary Table 3. Association between IL35 ligand and receptor expression in pancreatic carcinoma samples

		IL35 ligand					
		Low	High	Total	$\chi 2$	r	*P*
IL35	-	41	18	59	16.522	0.366	<0.0001
receptor	+	21	43	64			
	total	62	61	123			

Spearman rank correlation test
